# Differences in genome, transcriptome, miRNAome, and methylome in synchronous and metachronous liver metastasis of colorectal cancer

**DOI:** 10.3389/fonc.2023.1133598

**Published:** 2023-04-27

**Authors:** Josef Horak, Ondrej Kubecek, Anna Siskova, Katerina Honkova, Irena Chvojkova, Marketa Krupova, Monika Manethova, Sona Vodenkova, Sandra García-Mulero, Stanislav John, Filip Cecka, Ludmila Vodickova, Jiri Petera, Stanislav Filip, Veronika Vymetalkova

**Affiliations:** ^1^ Department of Molecular Biology of Cancer, Institute of Experimental Medicine of the Czech Academy of Sciences, Prague, Czechia; ^2^ Department of Medical Genetics, Third Faculty of Medicine, Charles University, Prague, Czechia; ^3^ Department of Oncology and Radiotherapy, Faculty of Medicine and University Hospital in Hradec Kralove, Charles University, Hradec Kralove, Czechia; ^4^ Institute of Biology and Medical Genetics, First Faculty of Medicine, Charles University, Prague, Czechia; ^5^ Department of Genetic Toxicology and Epigenetics, Institute of Experimental Medicine of the Czech Academy of Sciences, Prague, Czechia; ^6^ The Fingerland Department of Pathology, University Hospital in Hradec Kralove, Hradec Kralove, Czechia; ^7^ Biomedical Centre, Faculty of Medicine in Pilsen, Charles University, Pilsen, Czechia; ^8^ Unit of Biomarkers and Susceptibility, Oncology Data Analytics Program (ODAP), Catalan Institute of Oncology (ICO)-Oncobell Programme, Bellvitge Biomedical Research Institute Oncobell Programme, Bellvitge Biomedical Research Institute (IDIBELL), Hospitalet de Llobregat, Barcelona, Spain; ^9^ Program in Molecular Mechanisms and Experimental Therapy in Oncology (Oncobell), Oncobell Programme, Bellvitge Biomedical Research Institute (IDIBELL), Hospitalet de Llobregat, Barcelona, Spain; ^10^ Department of Surgery, Faculty of Medicine and University Hospital in Hradec Kralove, Charles University, Hradec Kralove, Czechia

**Keywords:** colorectal cancer, MiRNAome, methylome, transcriptome, liver metastasis

## Abstract

Despite distant metastases being the critical factor affecting patients’ survival, they remain poorly understood. Our study thus aimed to molecularly characterize colorectal cancer liver metastases (CRCLMs) and explore whether molecular profiles differ between Synchronous (SmCRC) and Metachronous (MmCRC) colorectal cancer. This characterization was performed by whole exome sequencing, whole transcriptome, whole methylome, and miRNAome. The most frequent somatic mutations were in *APC, SYNE1, TP53*, and *TTN* genes. Among the differently methylated and expressed genes were those involved in cell adhesion, extracellular matrix organization and degradation, neuroactive ligand-receptor interaction. The top up-regulated microRNAs were hsa-miR-135b-3p and -5p, and the hsa-miR-200-family while the hsa-miR-548-family belonged to the top down-regulated. MmCRC patients evinced higher tumor mutational burden, a wider median of duplications and deletions, and a heterogeneous mutational signature than SmCRC. Regarding chronicity, a significant down-regulation of *SMOC2* and *PPP1R9A* genes in SmCRC compared to MmCRC was observed. Two miRNAs were deregulated between SmCRC and MmCRC, hsa-miR-625-3p and has-miR-1269-3p. The combined data identified the IPO5 gene. Regardless of miRNA expression levels, the combined analysis resulted in 107 deregulated genes related to relaxin, estrogen, PI3K-Akt, WNT signaling pathways, and intracellular second messenger signaling. The intersection between our and validation sets confirmed the validity of our results. We have identified genes and pathways that may be considered as actionable targets in CRCLMs. Our data also provide a valuable resource for understanding molecular distinctions between SmCRC and MmCRC. They have the potential to enhance the diagnosis, prognostication, and management of CRCLMs by a molecularly targeted approach.

## Introduction

Metastasis, the process of spreading cancer cells from the primary site to distant organs, is a major cause of cancer mortality (1). The liver is the most common site of distant metastasis in colorectal cancer (CRC) patients. Colorectal cancer liver metastases (CRCLMs) are rather frequent. Even during the disease follow up, about 50% of CRC patients experience tumor occurrence at a distant site, resulting in a poor prognosis with a 14% five-year survival rate (2).

When the CRCLMs are not treated, the disease exhibits an unfavorable prognosis with the median overall survival (mOS) of less than 12 months. Even with the use of aggressive treatment (e.g. oxaliplatin-based treatment), the overall survival (OS) does not exceed 13-18 months (3–5). The only curative approach at present is surgical resection of isolated liver metastases. However, only 20% of resected patients attain long-term remission [1% of patients would develop liver metastasis during one-year follow-up, whereas 15% during five-year follow-up (5–11)], while 60-70% of patients experience local or distant recurrence (12). However, it remains clinically impossible to predict which patients are more likely to develop distant recurrence after resection of primary cancer. As the incidence and mortality of CRCLMs remain high, it is important and urgent to identify their etiology, molecular mechanisms, and biomarkers for early prediction and personalized treatment.

Patients with CRCLMs form a heterogeneous group. We can distinguish between Synchronous (SmCRC) and Metachronous (MmCRC) colorectal cancers. There is, however, still no consensus on the definition of SmCRC and MmCRC as used in the context of CRCLMs. Although by definition, all metastases are synchronous (occult or detectable at diagnosis), most definitions include detection at or before diagnosis or surgery of the primary tumor (13), while some also include metastases detected up to 3 (14, 15), 4 (16) or 6 months (17, 18) following diagnosis. It was suggested that both diseases might represent a distinct phenotype and impact therapy outcome, such as patients with SmCRC compared with patients with MmCRC have more adverse prognostic features and significantly shorter time to treatment failure (19). However, several studies did not confirm that (20, 21).

Over the past few decades, numerous preclinical and clinical studies have been conducted to uncover the underlying mechanisms of CRCLMs formation. However, only a limited number of results from these studies reached clinical practice, such as assessing PD-L1 expression in tumor cells using immunohistochemistry to provide prognostic information and predict response to treatment with PD-1/PD-L1 inhibitors (22). The lack of biomarkers in clinical practice is mainly because most studies focused on a single gene or a primary tumor.

In the present study, the high-throughput approaches with comprehensive bioinformatic analysis provided us with a platform for the analysis of metastatic tissue, both SmCRC and MmCRC, to find relevant hub genes and pathways and understand the molecular mechanism and characteristics of CRCLMs. In addition, we also investigated clinicopathologic information of SmCRC and MmCRC patients to improve patients´ management and follow-up.

## Materials and methods

### Sample collection, transport, and storage

In this prospective single-center study, histologically confirmed CRCLM patients were recruited at the Department of Oncology and Radiotherapy (Hradec Kralove, Czech Republic) between June 2019 and December 2021. All CRCLM cases were monitored regularly until May 31st, 2022. Patients with any personal history of previous other malignancy or with CRC-associated well-defined inherited syndromes (including Lynch syndrome, familial adenomatous, and MUTYH-associated polyposis) were excluded from the study. In total, 10 CRCLM patients were included in the study.

Biological samples were immediately put in an RNA Later stabilization reagent (Invitrogen, USA) and stored at − 80°C. Clinical data were collected from all subjects recruited in the study. The clinicopathological data for the patients recruited are reported in **Table 1**.

**Table 1:** Patients´ characteristics.

Characteristics		N=10	Synchronous (N=7)	Metachronous (N=3)
**Age**	**Years ± SD**	64.6 ± 8.1	69.9 ± 4.2	62.4 ± 8.5
**Gender**	**Men** **Women**	7 3	5 2	2 1
**Localization**	**Colon** **Rectum**	5 5	4 3	1 2
**Laterality^1^**	**Right-sided** **Left-sided**	2 8	2 6	1 2
**Histology**	**Adenocarcinoma, NOS** **Mucinous adenocarcinoma**	9 1	6 1	3 0
**Neoadjuvant therapy^2^**	**Yes** **No**	2 8	2 5	0 3
**Adjuvant therapy^3^**	**Yes** **No**	5 5	4 3	1 2
**Relapse after liver surgery**	**Yes** **No**	9 1	6 1	3 0
**Reoperation feasible**	**Yes** **No** **NA**	3 6 1	2 4 1	1 2 0
**Palliative therapy after liver surgery**	**Yes** **No** **NA**	5 4 1	3 3 1	2 1 0
**Living status**	**Alive** **Dead**	6 4	4 3	2 1

^1^Right- and left-sided primary tumors were defined as having their origin proximally or distally from the distant third of the transverse colon, respectively. ^2^Chemotherapy and/or targeted therapy before CRCLM resection. ^3^Chemotherapy after CRCLM resection. SD, standard deviation; NOS, not otherwise specified; NA, not applicable.

The local ethics committees at the Faculty Hospital in Hradec Kralove, Czech Republic (number of approval 201207- S01P) and the Institute of Experimental Medicine, Prague, Czech Republic (number of approval 2018/05) approved the study. All patients provided written informed consent.

The colon mucosa from healthy individuals the fresh frozen samples were collected consecutively during the planned colonoscopy and were described in Jungwirth et al. (23).

### Seed and soil mechanism and correction of bioinformatical data

The first cells invading the liver came from the primary CRC tumor. They thus also carry the genome/transcriptome of the primary tumor, respectively colorectum. Thus, comparing the genetic/molecular background of CRC cells with non-tumor liver cells could lead to results focused on organ specificity rather than on the distinctiveness of the metastasis relative to the healthy tissue. For this reason, we included a set of control colon tissues into the analyses apart from the patients’ paired samples (metastases and adjacent liver tissue). Overall, control colorectal tissues (n=10) were used to eliminate false positive results from contamination of liver tissue. These tissues were used from healthy individuals with no previous or current cancer. This correction was used for DNA methylation assessment and RNA seq analysis. A more detailed description of this correction is explained in the bioinformatics section of the methods.

### DNA isolation, quality, and quantity analyses

DNA isolation, quality and quantity analyses were all performed according to the manufacturer’s instructions. For detailed description see the appendix.

### Whole exome sequencing (WES)

We enriched protein-coding DNA using Human Whole Exome kit v7 (Agilent, USA) according to the manufacturer´s instructions. For detailed description see the appendix.

The data curation was performed using the standard tools – FastQC, MultiQC (24) for quality control, Trimmomatic for data trimming (25), BWA for alignment (26), PicardTools for deduplication, GATK pipline for variant calling (27), PureCN for tumor purity and CNV calling (28), SnpEff for variant annotations (29). For detailed description see the appendix. TMB was calculated as aTMB and fTMB as described in Zou et al. (30).

### DNA methylation analysis

DNA methylation analyses were done by the Infinium Methylation EPIC Kit (Illumina, USA) according to the manufacturer’s protocol and as described in Honkova et al. (31)

Raw data were processed with the minfi package (32). Data were normalized using the quantile method. A series of filtering was performed(probes with SNPs and crossreactive probes (33), probes to eliminate false positive results from tissue bias), resulting in the final number of 559,364 probes. SVA package was used for batch corrections (34). Differentially methylated loci were identified using limma package (35) and for the multiple testing of the false discovery rate (FDR), the p-values for the contrast of interest were adjusted to be below <0.01, which is regarded to be the most appropriate for microarray analysis (36). Annotatr package was used for the probe annotations (37) and clusterProfiler (38) and ReactomePA (39) for the functional analyses. For detailed description see the appendix.

### RNA isolation, quality, and quantity analyses

RNA isolation, quality and quantity analyses were all performed according to the manufacturer’s instructions. For detailed description see the appendix.

### RNA-seq analysis

Ribosomal RNAs (rRNAs) were removed using NEBNext rRNA Depletion Kit (Human/Mouse/Rat, New England Biolabs, USA). RNA sequencing cDNA libraries were constructed according to the NEBNext Ultra II Directional RNA Library Prep Kit for Illumina, as provided by the manufacturer (New England Biolabs, USA). For detailed description of sequencing see the appendix.

The data curation was performed using the standard tools – FastQC, MultiQC for quality control (24), Trimmomatic for data trimming (25), BBmap for ribosomal RNA filtering, STAR for alignment (40), RSEM for quantification (41). The tumor purity, immune and stromal proportion, and contamination of liver tissue were assessed by the ratio of somatic variants in non-differently expressed genes and by the ESTIMATE R package (42) ahead of the final expression calculation. The normalized counts of metastatic samples were then cleaned from the contamination of liver tissue by subtracting the calculated proportion of the liver tissue normalized counts. EdgeR was used for identifying differentially expressed genes (43). After Benjamin-Hochberg adjustment, gene was considered deregulated with FDR lower than 0.01 and absolute logFC greater than 1. culsterProfiler (38) and ReactomePA (39) were used for functional analyses. For detailed description see the appendix.

### miRNA isolation, quality, and quantity analyses

RNA isolation, quality and quantity analyses were all performed according to the manufacturer’s instructions. For detailed description see the appendix.

### Small RNA-sequencing

The next-generation sequencing library preparation was carried out as described in Sabo et al. (44) and Cervena et al. (45). MiRNA libraries were constructed using the NEB Next Multiplex Small RNA Library Prep Set for Illumina (New England BioLabs, USA) according to the manufacturer’s protocols.

FastQC and MultiQC (24) were used for quality control, Cutadapt tool (46) for trimming and BBMap for ribosomal RNA filtering. The miRge3.0 pipeline (47) was used for alignment and quantification. Significantly different miRNAs were identified by EdgeR R package (43). After BH adjustment, the miRNA was considered DE with a false discovery rate lower than 0.05.

### External validation

For external validation of our data, the GSE62321 set was used (48, 49). This set comprises pairs of primary tumors and hepatic metastases before chemotherapy from 13 patients.

### Bioinformatic in silico analysis

#### DepMap data analysis

To analyze the *IPO5* gene interactions, the correlation data were downloaded from the CRISPR (Avana) Public Depmap v20Q3 portal (https://depmap.org/portal/download/) for all cell lines in the database (1,078 cell lines). Gene correlation between *IPO5* knockout effect and gene expression was considered significant for a p-value lower than 0.05 and an absolute value of correlation coefficient higher than 0.1. Functional enrichment analysis of significantly correlated genes with the *IPO5* effect was performed using the clusterProfiler, and ReactomePA R packages. Only terms associated with a BH-adjusted p-value lower than 0.05 were considered.

## Results

### Patients´ characteristics

**Figure 1:**
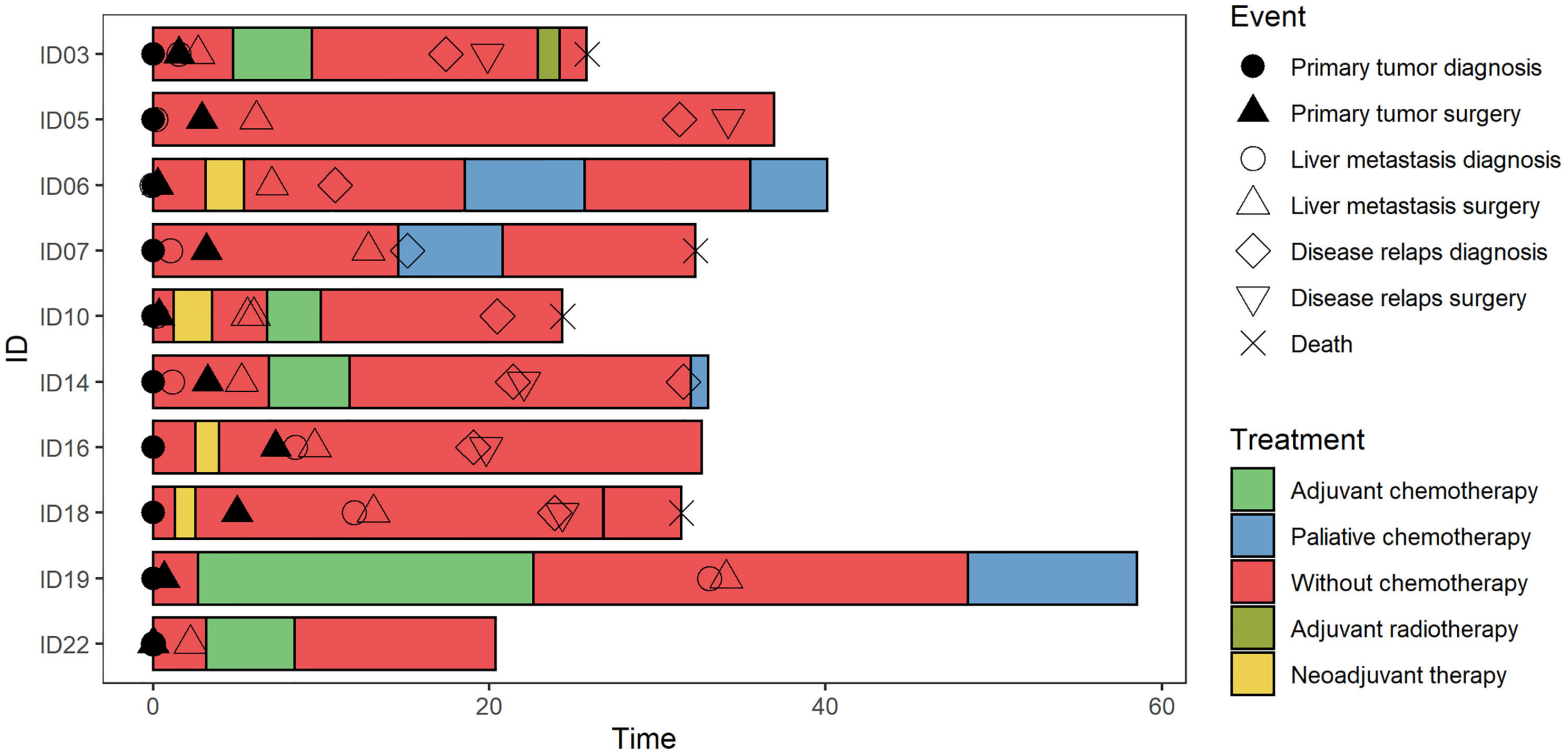
Swimmer plot. The clinical and pathological characteristics of 10 patients included in the study. Each bar represents one subject in the study, and symbols along each bar represent various relevant clinical events.

The clinical and pathological characteristics of 10 patients with liver metastasis included in the study are described in **Figure 1** and **Table 1**. Patients were predominantly males (70%) with a mean age of 64.6 ± 8.1 years. At the time of diagnosis, three patients had CRC stage III, while seven patients had stage IV disease with metastases confined to the liver. Regarding the therapy, two subjects received chemotherapy and/or radiation therapy prior to the surgical resection of the primary tumor and one after the primary tumor resection. All patients had CRCLM eligible for potential resection at the time of enrollment. In terms of chronicity, the group SmCRC patients comprised seven patients, while the MmCRC group three. Two patients received induction therapy with Bevacizumab/mFOLFOX6 prior to the CRCLM resection and six patients received a fluoropyrimidine-based adjuvant chemotherapy following CRCLM resection. Nine patients relapsed with a median relapse-free survival (RFS) of 12.0 months (range 2.3–26.5 months). Three of these patients were eligible for subsequent surgery and five received palliative fluoropyrimidine-based chemotherapy and/or targeted therapy. Four patients had died during the follow-up period with a median OS of 19.4 months (range 18.6–35.6 months).

### DNA-based analysis

#### Analysis of WES data

We have successfully analyzed the DNA isolated from 10 paired CRCLM and adjacent liver tissue samples.

After alignment, a range from 81 to 169 million properly aligned read pairs per sample were obtained. In summary, the percentage of adequately mapped read pairs reached 99% for all samples. The average read depth for each patient can be found in **Supplementary Table S1**. Due to low data quality control, two out of ten patient samples had to be discarded from this analysis.

With the advantage of robust WES data, several features, such as mutational analysis, tumor mutation burden and copy number variations were analyzed with obtained WES data. The first analysis of all samples was performed, and then stratification for either SmCRC or MmCRC was presented.

### Mutation analysis

WES in CRCLM and adjacent liver tissue DNA was performed to identify somatic tumor-specific single nucleotide variants (SNV) and short insertion/deletion (Indel) mutations characterizing liver metastasis.

In total, 1,900 heterozygous and homozygous SNVs and Indels (1,779 substitutions, 4 double substitutions, 58 short insertions, and 59 short deletions) have been discovered as different between these two groups: 104 with high, 361 with moderate, 645 with low and 816 with modifier putative effect. Regarding the SNV effect, the largest group of identified variants belonged to the missense and intron variants, followed by synonymous variants. For the Polyphen2 calculation, we filtered out only non-synonymous exonic variants, and the results were as follows: benign prediction was among all samples in the range 32-48%, possibly damaging in the range 17-33%, and damaging prediction in the range 28-48%. After stratification for chromosome localizations, the observed variants’ distribution was similar across all chromosomes (**Supplementary Figure 1**).

**Figure 2:**
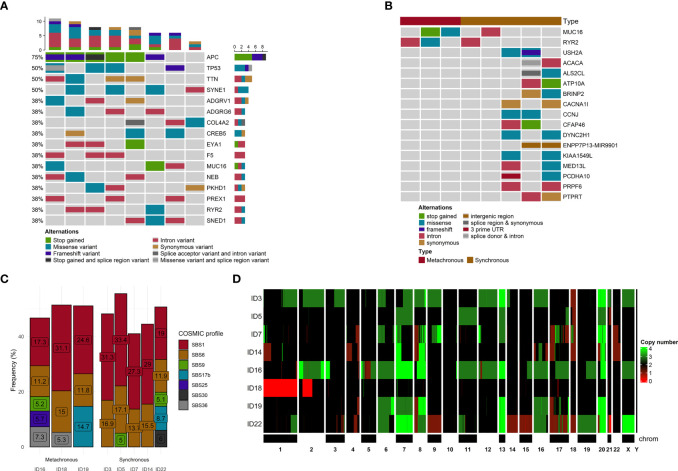
Mutational and copy number variants analyses. **(A)** The distribution of genetic alterations detected in all samples. Only genes affected, at least in 3 samples, are pictured. Each column represents a patient, and each row represents a gene. Different colors indicate different mutation types. The bar chart on the top shows the total number of the given gene mutations observed in the sample. **(B)** The distribution of genetic alterations detected between SmCRC and MmCRC patients. Each column represents either SMCRC or MmCRC group of patients, and each row represents a gene. Only genes with differences in relative group distribution difference of 50% or higher are pictured. Different colors indicate different mutation types. **(C)** The distribution of mutational signatures in percents according to the COSMIC SBS V3 database across all samples. Only signatures with distribution in single sample of more than 5% are pictured. Each column represents a patient. **(D)** Heatmap of CNV distribution across all samples. Each row is represented by a patient. A chromosome represents each column. The green color represents a gain, while red represents a loss within a chromosome locus.

The most frequent somatic mutations at the time of enrolment were in *APC* (75%), *SYNE1* (50%), *TP53* (50%), and *TTN* (50%) genes. Only one *APC* mutation (rs121913333, c.2680C>T, p.Arg894*) was detected more than once (patients ID 5 and 22, **Supplementary Table S2**). No other mutations were observed more than once. The top genes with a mutation frequency of > 38% are presented in **Figure 2A**.

In the terms of chronicity, we further compared the mutational landscape between SmCRC (n=5) and MmCRC (n=3) patients. No significant differences in distribution among these two groups were noticed after stratification for putative effect, SNV effect, chromosome distribution, or Polyphen2 characterization (**Supplementary Figure 2**).

Using the OncoPrint for visualization of differences in the distribution of genomic variations, the most observed difference between these two groups was noticed for *MUC16, RYR2* (SmCRC in 60% vs MmCRC 0%) and *USH2A, ACACA, ALS2CL, ATP10A, BRINP2, CACNA1l, CCNJ, CFAP46, DYNC2H1, ENPP7P13, KIA1549L, MED13L, PCDHA10, PRPF6, PTPRT* (0% vs 66%) genes. No other substantial differences were observed between these two groups (**Figure 2B**).

### Tumor mutation burden analysis

The overall TMB representing the number of single nucleotide variations (SNVs), multiple nucleotide variations (MNVs), and InDels per Mb was calculated. TMB was defined as aTMB, which includes synonymous mutations, and fTMB, which excludes synonymous mutations. The average value of aTMB was 5.99 in all patients, and the median value of aTMB was 6.62 in all patients (SmCRC aTMB mean 5.84 and median 6.60, MmCRC aTMB mean 6.22 and median 6.64, respectively). The average value of fTMB was 5.21 in all patients, and the median value of aTMB was 5.75 in all patients (SmCRC fTMB mean 4.98 and median 5.59, MmCRC fTMB mean 5.59 and median 6, respectively).

### Mutational signature

Mutational signature analysis revealed that transition mutations were more common than transversion mutations, and C > T substitutions were predominant in our samples (**Supplementary Figure 3**).

To better understand the pathogenesis of CRCLM, we performed mutational signature analysis on 2,696 (1,779 heterozygous/homozygous and 917 subclonal) SNVs by analyzing the six mutation classes (C > T, C > A, T > C, T > G, C > G, and T > A). According to the COSMIC SBS V3 database, we identified eight mutational signatures with a higher than 5% proportion in our CRCLM samples (in descending order SBS1, SBS6, SBS17b, SBS36, SBS30, SBS25, SBS3, and SBS9, **Figure 2C**).

In the terms of chronicity, SmCRC patients had a higher proportion of SBS1 (cause of mutations is age related) and SBS6 (cause is related to defective mismatch repair) mutational signatures, while MmCRC patients displayed a more heterogeneous distribution of mutation signatures. Given the small number of patients in each group taking this result with caution is necessary.

### CNV analysis

**Figure 3:**
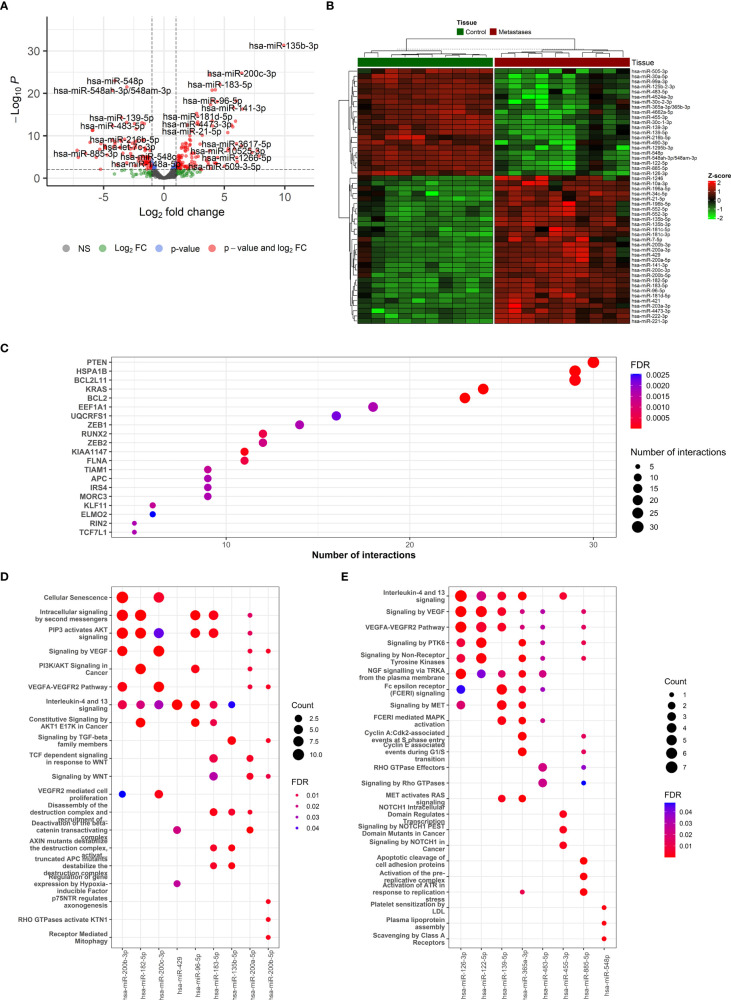
The description if miRNA-based results and functional enrichment of its targets. **(A)** Volcano plot reporting the log2FC and the adjusted p-value in the DE analysis between metastasis and adjacent liver tissue. Thresholds: adjusted p-value < 0.0+ and |log2FC| > 1. **(B)** Heatmap of top 50 differentially expressed miRNAs between metastasis and adjacent liver tissue. **(C)** The top miRNA targets from target enrichment analysis. **(D)** The Reactome enrichment analysis of the top 10 differentially up-regulated miRNAs. **(E)** The Reactome enrichment analysis of the top 10 differentially down-regulated miRNAs.

Using PureCN, we identified recurrent CNVs affecting 5,432 genes (4,564 gained genes and 868 lost genes, **Supplementary Table S3**) within at least 50% of our group, containing several already known and putative driver genes (**Figure 2D**). For all samples, the median duplication length was 2,675,594 bases, while the median deletion length was 783,537. The density of genomic sizes of structural variants in all CRCLM patients is depicted in **Figure 3C**. The deletions ranged from ~239 b to 92 Mb, with a distinct peak at ~496 kb. A broad range of differently sized duplications (~264b-147Mb) was observed, with a peak at 1.2 Mb.

In the terms of chronicity, for SmCRC, the median duplication length was 2,166,142 (range 264b-147Mb, peak at 1.2Mb) bases while the median deletion length was 116,0517 (range 241b-92Mb, peak at 929kb). For MmCRC, median duplication length was 3,267,214 and median deletion length 607,852 (range 269b-125Mb, peak at 1.6Mb, respectively 239b-66Mb, peak at 382kb, **Supplementary Tables S4** and **Supplementary Figure 4**).

### DNA methylation analysis

We have successfully analyzed the DNA isolated from 8 paired CRCLM and adjacent liver tissue samples.

To obtain an overview of DNA methylation profiles, first, the principal component analysis based on the differential DNA methylation of the CpG loci showed a clear separation among CRCLM and adjacent liver tissue (**Supplementary Figure 5**).

We next analyzed the major differences in DNA methylation distribution and found 25,865 CpG sites differentially methylated in CRCLM tissue compared to adjacent liver tissue. Among the differentially methylated CpG sites, 5,185 were hypermethylated and 20,680 hypomethylated and mapped to 3,412 different genes (1,043 hypermethylated and 2,369 hypomethylated). Hypermethylated CpG sites were predominantly observed in CpG islands (64.4%). On the other hand, hypomethylated CpG sites were mainly located in intergenic regions, the so-called open sea (79.8%) (**Supplementary Figure 5**).

**Figure 4:**
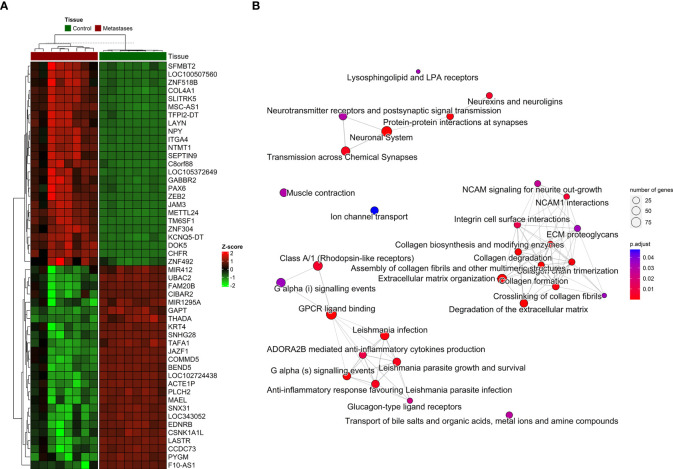
The distribution of DNA methylation patterns. **(A)** Heatmap of top 25 significantly hypermethylated and top 25 significantly hypomethylated genes between metastasis and adjacent liver tissue. **(B)** Emapplot of the overrepresentation analysis (ORA) pathway enrichment according to the Reactome database.

Additionally, we identified those genes with the highest quantitative differences in methylation between CRCLM and adjacent tissue. The comparison was based on the analyzed Δβ-values and CpG sites with an adjusted p-value < 0.01. The distribution of the top 25 genes with the highest differences in methylation and hierarchical clustering is depicted in **Figure 4A**.

For the visualization, interpretation and analysis of affected pathways, the Reactome database was used for pathway enrichment in our list of differentially methylated genes (**Supplementary Table S5** and **Figure 4B**). The hypomethylated genes were involved in processes such as Cell adhesion and extracellular matrix (ECM) organization, and the hypermethylated genes in Neuroactive ligand-receptor interaction or calcium signaling pathways and various cancer-related pathways.

### RNA-based analyses

#### RNA-seq analysis

We have successfully analyzed the RNA isolated from 9 paired CRCLM and adjacent liver tissue samples and 9 non-paired normal colon tissue samples.

The expression profiling of transcripts and differential expression of genes between the CRCLM and adjacent liver tissue samples were analyzed using RNA-seq data. As for WES, the first analysis of all samples was performed, and then stratification for either SmCRC or MmCRC was presented.

**Figure 5:**
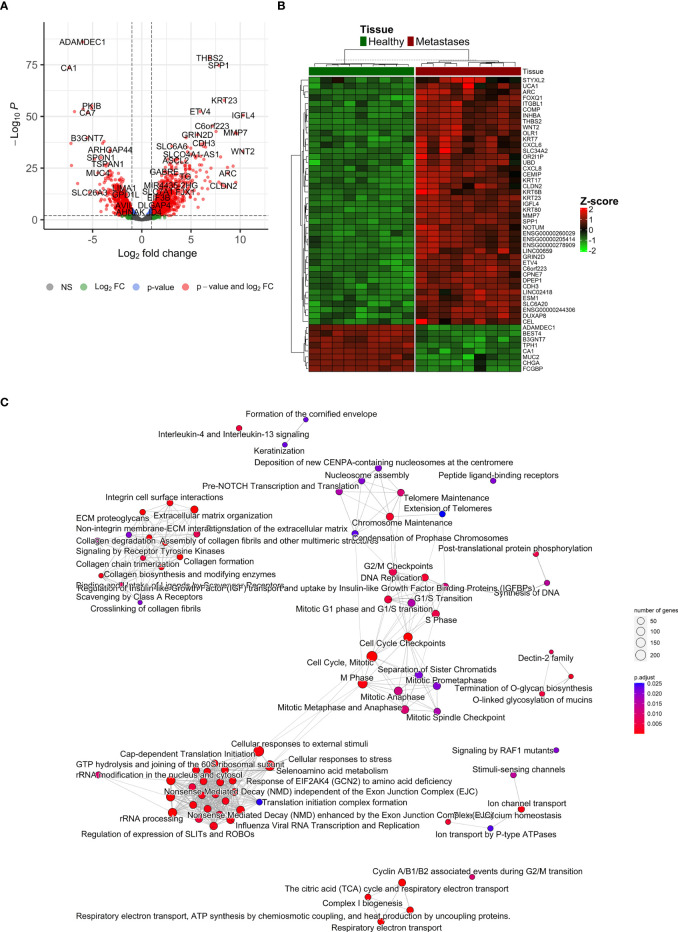
Differentially expressed genes ‘characterization. **(A)** Volcano plot reporting the log2FC and the adjusted p-value in the DE analysis between metastasis and adjacent liver tissue. Thresholds: adjusted p-value < 0.01 and |log2FC| > 1. **(B)** Heatmap of top 50 differentially expressed genes between metastasis and control colon tissue. **(C)** Emapplot of the gene set enrichment analysis (GSEA) of the significantly deregulated pathways in the Reactome terms (adj. p-value < 0.05, top 80 terms).

After the normalization of liver contamination in metastatic tissue by using paired liver samples, 14,121 genes remained in the analysis. A total of 2,711 differentially expressed genes (DEGs, adjusted p < 0.01 and absolute value of logFC > 1) were identified by an analysis of CRCLM compared with colon tissue. Of these, 1,719 were up-regulated, and 992 were down-regulated in CRCLM samples (**Figure 5A** and **Supplementary Table S6**). The stratification for the top 50 significant DEGs is depicted in **Figure 5B**.

To further characterize the changes in mRNA expression levels in CRCLM and adjacent liver tissue samples, functional pathway enrichment analysis was performed to identify the main related biological functions. Gene set enrichment analysis (GSEA) uncovered 124 significantly deregulated pathways in the Reactome terms (adj. p-value < 0.05, top 80 in **Figure 5C**). Overrepresentation analysis (ORA) of up-regulated (logFC > 1) and down-regulated (logFC < -1) genes are reported in **Supplementary Table S7**. The most significant results were obtained for down-regulated genes, which were enriched in Reactome terms related to Ion channel transport, the citric acid cycle, and respiratory electron transport. Conversely, the up-regulated genes were enriched prevalently in ECM organization and degradation of ECM. Similarly, GSEA on KEGG terms uncovered 27 significant terms, ORA on upregulated genes described 15 significant KEGG terms, and ORA on downregulated genes described 17 significant KEGG terms (**Supplementary Figure 6**).

In the terms of chronicity, only two genes were significantly down-regulated in SmCRC compared to MmCRC (*PPP1R9A* logFC=2.8, FDR=0.05, and *SMOC2* logFC=2.6, FDR=0.05).

### miRNA-seq analysis

We have successfully analyzed the miRNA isolated from 10 paired CRCLM and adjacent liver tissue samples.

To identify the miRNA signature for CRCLM detection, the miRNA expression profiling between the CRCLM and adjacent liver tissue sample was analyzed using miRNA-seq data. As for WES and RNA-seq, the first analysis of all samples was performed, and then stratification for either SmCRC or MmCRC was presented. Additionally, we also looked at valid miRNA targets that may be associated with differential miRNA expression levels in CRCLM.

After sequencing, an average of 57.3% of the reads were aligned to miRNA sequences, while 10.2% were aligned to other small non-coding RNAs (sncRNAs) (**Supplementary Figure 7**; **Supplementary Table S8** **).** In total, 1,603 miRNAs were detected, with an average of 845 miRNAs detected in each sample and 670 miRNAs with sufficient coverage across all samples for differential expression calculation. Other classes of sncRNAs were seen, including, tRNAs, and snoRNAs. The distribution of the detected non-miRNA sncRNAs in the sample group is shown in **Supplementary Table S9**. In total, 2,415 non-miRNA sncRNAs were identified in all subject groups.

In total, 123 miRNAs were up- and 92 down-regulated in CRCLM compared to adjacent liver tissue (**Supplementary Table S10**). Among the up-regulated top miRNAs were hsa-miR-135b-3p, hsa-miR-200c-3p, hsa-miR-200b-5p, hsa-miR-183-5p, hsa-miR-182-5p, hsa-miR-200b-3p, hsa-miR-96-5p, hsa-miR-135b-5p, hsa-miR-429 and hsa-miR-200a-5p. Among the top down-regulated were hsa-miR-548p, hsa-miR-99a-3p, hsa-miR-548ah-3p/548am-3p, hsa-miR-139-5p, hsa-miR-125b-2-3p, hsa-miR-30c-1-3p, hsa-miR-455-3p, hsa-miR-126-3p, hsa-miR-483-5p, hsa-miR-365a-3p/365b-3p. The levels of the most significant deregulated miRNAs are reported in **Figures 3A, B**.

In the terms of chronicity, only two miRNAs were deregulated between SmCRC and MmCRC (hsa-miR-625-3p and hsa-miR-1269-3p, **Supplementary Table S11**).

The miRNA binding to its target transcript does not necessarily lead to a downregulation of gene expression. In fact, most of the observed miRNA binding events have minor functional consequences. Thus, focusing on miRNA binding alone has limited value in predicting valid miRNA targets, i.e., downregulated targets. To alleviate this problem, we directly determined the target downregulation of miRNA-seq with RNA-seq. Among the top miRNA targets were observed *PTEN, HSPA1B, BCL2L11, KRAS*, and *BCL2* genes (**Figure 3C**). Gene set enrichment analysis of targeted mRNAs by miRNAs can support the role of differentially expressed miRNAs. The Reactome enrichment in **Figures 3D, E** and the KEGG enrichment analysis of differentially expressed miRNA-targeted pathways is presented in **Supplementary Figure 7**. Among these pathways Signaling pathways regulating pluripotency of stem cells, TGF-Beta, WNT, Ras, mTOR, and NOD-like receptor signaling pathway were the most prominent pathway targeted by deregulated miRNAs.

### Integration of results from the genome, methylome, transcriptome, and miRNAome analyses

**Figure 6:**
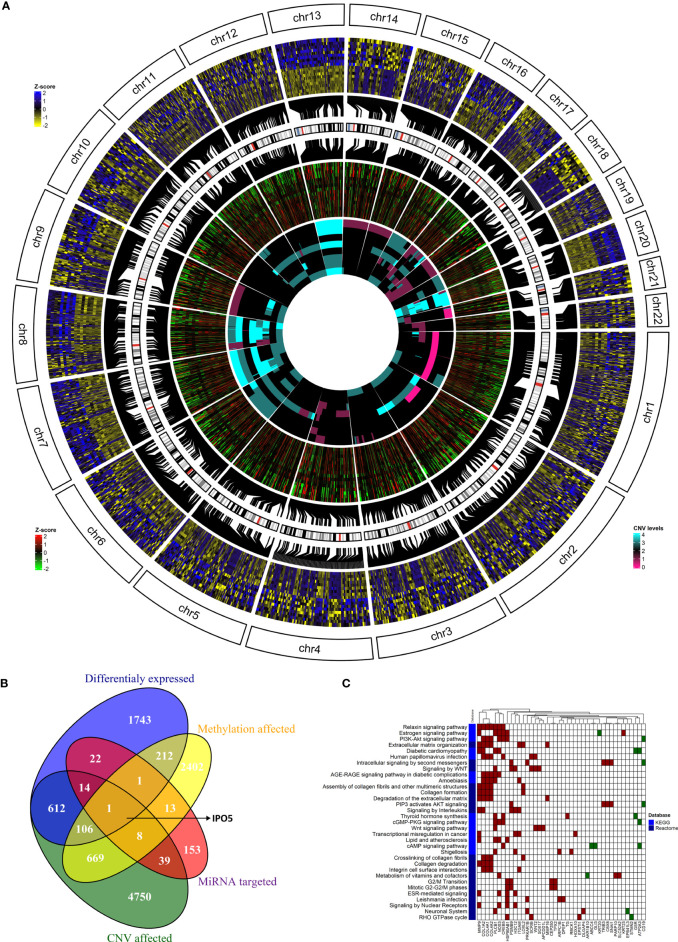
The integration of results from the genome, methylome, transcriptome, and miRNAome analyses. **(A)** The overlap of alterations discovered between the CRCLM patients across all platforms. The outside heatmap circle represents the significant DEGs, the blue color represents up-regulation, yellow color represents down-regulation. The middle heatmap circle represents the methylation level for the genes depicted in the outside circle, the red color represents hypermethylation, the green color hypomethylation, and the black color non-significant changes. The inner heatmap represents the CNV level for the genes depicted in the outside circle. The cyan color represents gains, the purple represents losses. **(B)** Venn diagram showing the overlap between DEGs obtained in the analysis stratified by methylation profile, CNV, and miRNA expression levels. **(C)** The heatmap of KEGG (light blue) and Reactome (dark blue) enriched pathways and genes from integrated analysis of genome, methylome and transcriptome analyses. Up-regulated genes have red square while down-regulated genes have green square. White square means that the gene is not present in the specific pathway.

To go behind the source of gene deregulation in our set, we also performed integrated analysis for the regulatory relationships of DNA methylation, genetic selection, and miRNA expression data, together with mRNA expression data. The overlap of alterations discovered between the CRCLM across all platforms was performed (**Figure 6A**). We have identified 2,711 differentially DEGs, out of which 612 could be explained as deregulation due to the CNV effect, 22 due to the miRNA expression levels, and 212 due to methylation profile. CNV, methylation, miRNA, and DEGs overlapped at one gene, *IPO5* (high expression profile, high miRNA expression, hypomethylation profile, and gain in the CNV analysis, **Figure 6B**).

To analyze the *IPO5* gene interactions, the correlation data were downloaded from the CRISPR (Avana) Public Depmap v20Q3 portal (https://depmap.org/portal/download/) for all cell lines in the database (1,078 cell lines). With the threshold for adjusted p-value 0.05 and absolute value of correlation coefficient >0.1, 1,739 genes that interact with the *IPO5* gene were identified.

The most significant results obtained for *IPO5* gene enriched in Reactome were related to the cell cycle. The Reactome analysis for *IPO5* gene was visualized in **Supplementary Figure 8** and **Supplementary Table S12**.

It is well known that one miRNA can regulate several genes and vice versa, multiple miRNAs can regulate one gene. For this reason, we were also interested in overlapped genes without considering the miRNA expression levels. This combined analysis resulted in 107 genes (**Figure 6B** and **Supplementary Table S13**). GSEA on Reactome and KEGG terms uncovered significant terms (**Figure 6C** and **Supplementary Table S14**) related to relaxin, Estrogen, PI3K-Akt, WNT signaling pathways and intracellular signaling by the second messenger.

Due to the low numbers in each group, no stratification for SmCRC and MmCRC was not performed.

### External validation

To validate our results, the GSE62321 set was used (48, 49). After data processing 9,115 genes were left out of which 2,873 genes were significantly deregulated between colon and metastases. The intersection between our and this validation set resulted in 5,918 genes where 592 genes were significantly deregulated (332 up- and 260 down-regulated) in the validation set and 1,060 (624 up- and 436 down-regulated) in our set. The final intersection between these two sets resulted in 366 DEGs where 204 were up- and 162 down-regulated (**Supplementary Table S15**).

The same validation set also comprised primary tumors from patients with metastasis (105 significantly DEGs between primary tumor and metastasis). Comparing this set with our set, the intersection resulted in 9 significant DEGs (2 upregulated and 7 downregulated, **Supplementary Table S16**).

## Discussion

In newly diagnosed CRC patients without metastases, elucidating potential molecular risk factors for the development of liver metastasis is paramount, as they could have important clinical implications. For this reason, we focused on CRCLM patients as, after all, metastasis constitutes the primary cause of death for >90% of patients with CRC (1). Understanding the dynamics of this process will help identify targets for molecular therapies that may halt or possibly reverse cancer growth and metastasis. By identifying these targets, therapies can be designed to target cancer cells more precisely through either selective disruption of pathways necessary for cancer cell survival/growth or artificial modulation of the patient’s immune system to generate a response against cancer cells. Besides, given its potential impact on patient care, a better understanding of the development of distant metastases is critical.

This study aimed to molecularly characterize metastatic tissues in CRCLM patients and explore whether molecular profiles differ between SmCRC and MmCRC. This characterization was performed by high-throughput approaches, such as WES, whole transcriptome, whole methylome, and miRNAome and analyzed by comprehensive bioinformatic tools. We hypothesize that combining all these approaches into comprehensive molecular profiling would lead to more relevant findings than single analyses published previously. We have demonstrated the utility of this integrative and broad approach where each genomic platform, independently and in combination, contributed to detecting pathogenic variants in all samples. We first analyzed each approach separately and then combined all the analyzes together and summarized the results below.

By analyzing the results from the WES only, we revealed an *APC* mutation (rs121913333, c.2680C>T, p.Arg894*) that was detected more than once. No other mutation was observed in any other gene more than once. Similarly, Naxerova and co-authors reported that in 65% of cases, regional lymphatic and distant metastases arose from independent subclones from the primary tumor (50).

CRC cells acquire a capacity to evade the primary CRC through morphological changes such as EMT, migration through the ECM, invasion into the neighboring tissues, intravasation, survival in the circulation, extravasation and finally, colonization to distant liver forming more aggressive CRCLMs (51). The ability of cells to undergo all these steps in the metastatic cascade requires them to acquire specific characteristics connected to the ‘hallmarks of cancer’. In addition, Vermeulen et al. (52) proposed liver metastasis as a heterogeneous tumor and classified CRC liver metastasis into 3 growth patterns, i.e., pushing, desmoplastic, and replacement, based on histological differences. This agrees with our findings, the up-regulated genes in our study were prevalently enriched in the ECM degradation and organization, and hypomethylated genes were involved in processes such as Cell adhesion and ECM organization.

Deregulation of gene expression in the metastatic process is a complex process involving several modalities, including genomic changes or epigenetic modifications. Although the mutations in a few driver genes, such as *APC* and *TP53*, are shown to be one of the drivers of CRC progression as well as a biomarker of CRC stage and resistance to various CRC therapies (53), the true heterogeneity between patients is represented by the CNV distribution. The differences between patients and even the tumor cells were observed as highly heterogeneous (54). Our results supported these findings, as only a relatively low number of genes was mutated in our cohort while CNV distribution affected more genes, and the range was higher. Although only 10% of affected genes by CNV had an impact on expression deregulation, it was the major source of gene deregulation in our set. The well-known gains and losses occurring in the CRC progression, specifically on chromosomes 8, 13, 20, and chromosomes 4 or 18, respectively (55) were also observed in our cohort.

Another gene expression deregulation source are small non-coding RNAs, such as miRNAs. In this study, among the up-regulated top miRNAs were hsa-miR-135b-3p and -5p, and the hsa-miR-200-family. Among the top down-regulated was hsa-miR-548-family. Serum hsa-miR-135b (56) and has-miR-200c (57) levels were already considered as diagnostic (56) or prognostic and metastasis-predictive (57) biomarkers in patients with CRC. High serum miR-200c demonstrated a significant positive correlation with lymph node metastasis, distant metastasis, and prognosis (57). Regarding the has-miR-548-family, has-miR-548b was already found to be down-regulated in CRC patients as well as in CRC cells and even lower in advanced stages. The overexpression of miR-548b suppressed cells’ proliferation and induced apoptosis. Our observation means that the downregulation of miR-548b may lead to uncontrolled cell division. Besides, WNT2 was predicted to be the downstream gene binding miR-548b (58). Almost all CRC cases demonstrated hyperactivation of the WNT pathway, which is considered to be the initiating and driving event of CRC (59).

When the intersection of all our data is considered, the combined data from CNV analysis, DNA methylation profiles, and mRNA sequencing led to identifying the IPO5 gene. Several sources upregulated the *IPO5* gene: in 75% of our samples, the gain in this gene was observed and concurrently was significantly hypomethylated. On the other hand, miRNAs targeting *IPO5* were upregulated as well. We hypothesize that the upregulation of targeting miRNAs might be driven by counter-regulation to reduce the impact of the *IPO5* upregulation to establish a balanced state. The *IPO5* gene belongs to the Karyopherins family, which are crucial regulatory molecules of nuclear plasma transport and represent the most classic cellular transporter proteins, including both importins and exportins (60, 61). Transport proteins with a molecular weight greater than 40 kDa help to transport molecules between the cytoplasm and the nucleus through the nuclear pore complex, such as transcription factors, splicing factors, and other proteins (62, 63). Therefore, karyopherin dysfunction can lead to altered transport activity and cause abnormal localization of oncogenic factors, consequently leading to tumorigenesis (64). Recently, Zhang et al. (65) noticed that the expression of the *IPO5* gene was gradually growing with the increasing CRC stage. In addition, the high *IPO5* expression, especially in CRC cells, was also confirmed in the TCGA database and Oncomine database in their study. Besides, the functional assays revealed that *IPO5* promoted CRC growth *in vitro* and *in vivo* through the RASAL2 nuclear translocation, followed by the activation of the RAS signaling pathway. Inhibition of the nuclear transport system, therefore, has future potential for therapeutic intervention and could contribute to the elucidation of disease mechanisms. *IPO5* gene also seems to play an important role in the metastatic process *via* matrix metalloproteinase 7 (*MMP7*) regulation (66) and EMT induction (67). Furthermore, *IPO5* expression may affect the tumor immune microenvironment and mediate tumor immune response (68). The commensal microbiota has already been shown to influence immunity as well as tissue development. In the context of cancer, commensal bacteria have been shown to play a key role in modulating tumor microenvironment, which drives cancer therapy responses (69). Therefore, direct inhibition of *IPO5* function may uncover a promising targeted therapeutic strategy for CRCLM.

On the other hand, as one miRNA can regulate several genes, and conversely, multiple miRNAs can regulate one gene, we were also interested in intersection regardless of miRNA expression levels. This combined analysis resulted in 107 deregulated genes. GSEA on Reactome and KEGG terms revealed significant terms related to relaxin, estrogen, PI3K-Akt, WNT signaling pathways, and intracellular second messenger signaling. All these pathways were assigned to the CRC evolution and thus made our results valid even if they were obtained from a smaller number of patients.

The occurrence of CRCLM, such as SmCRC vs. MmCRC detection, was mainly investigated and reported in the surgical case series (21, 70, 71). The gene expression and molecular patterns of SmCRC and MmCRC are considered different. Synchronous liver metastases are similar to local invasion and are more inclined to become a disseminated disease (72). One of our main aims was the comparison of the molecular characteristics of these two groups. MmCRC patients evinced higher TMB, a wider median of duplications and deletions, as well as heterogeneous mutational signatures in our set of patients. We thus hypothesize that these differences could be explained as a consequence of the adaptation of resistant clones to escape previously applied chemotherapy in MmCRC patients. Similarly, higher TMB in MmCRC patients could be related to better immunosurveillance and thus delayed relapse (compared to SmCRC), as higher TMB is associated with higher tumor immunogenicity (73). However, given the small number of patients in each group, it is necessary to take this result with caution.

Further, in terms of chronicity, we observed a significant down-regulation of SMOC2 and PPP1R9A genes in SmCRC patients compared to MmCRC. SMOC2 is an extracellular glycoprotein involved in a broad spectrum of cellular processes, including cell cycle, cell attachment and migration, angiogenesis, and others (74). It has a suppressive role in tumor growth, migration, colon and sphere formation role in CRC cells and could be considered a tumor suppressor in CRC progression (74). PPP1R9A gene codes a protein Neurabin-1 (75), which binds to protein phosphatase 1 (PP1) and inhibits its activity (76). Neurabins are highly concentrated in dendritic spines, and post-synaptic densities and their function is to regulate synaptic transmission in mammalian neurons (77). Despite there are no reports on the role of Neurabin-1 in carcinogenesis, the loss of Neurabin-2 (also known as Spinophilin or PPP1R9B), which has 80% homology in its sequence and similar biological functions to Neurabin-1, was associated with more aggressive histological phenotype, faster relapse, poor survival, and a low response to fluoropyrimidine-based chemotherapy in CRC (78, 79). Interestingly, the loss of Neurabin-2 is associated with an increase in the stemness properties (80) which may facilitate the spread to the liver. Given these circumstances, it could be hypothesized that the down-regulation of both *SMOC2* and *PPP1R9A* genes might be associated with rapid CRCLM formation in patients with SmCRC. Further research is warranted to evaluate the potential value of these genes as predictors of prognosis and risk of CRCLM formation in CRC patients.

Only two miRNAs were deregulated between SmCRC and MmCRC, hsa-miR-625-3p and has-miR-1269-3p. Interestingly, hsa-miR-625-3p induced oxaliplatin resistance by abrogating MAP2K6-p38-regulated apoptosis and cell cycle control networks (81). Bu et al. (82) observed that in stage II CRC patients, miR-1269a expression in their surgically removed primary tumors was strongly associated with the risk of CRC relapse and metastasis. The authors hypothesized that miR-1269a was a potential marker to contribute to adjuvant chemotherapy decisions for CRC patients and a potential therapeutic target to deter metastasis. The fact that we found these two miRNAs deregulated right between the SmCRC and MmCRC patients where the MmCRC patients had already undergone both surgical and adjuvant treatment strengthens our results considerably.

In summary, we have identified genes and pathways that may be considered actionable targets in CRCLMs (e.g. *IPO5* gene, hsa-miR-135b-3p and -5p, the hsa-miR-200-family, and hsa-miR-548-family, WNT and PI3K-Akt signaling). The data presented here also provide a valuable resource for understanding molecular distinctions between SmCRC and MmCRC, and have the potential to enhance the diagnosis, prognostication and management of CRCLMs by a molecularly targeted approach. The strength of this study lies in the sizeable high-throughput analysis, including the non-trial patients reflecting real-world patients. On the other hand, we are aware of certain limitations of our study, such as the lack of primary tumors due to the prospective design. However, the intersection between our and validation sets confirmed the validity of our results.

Detailed molecular analysis of mechanisms that mediate metastatic expansion to the liver will contribute to early detection and prevention. Future research should focus on elucidating the origin of CRCLM based on the molecular mechanisms and clinical characteristics – this elucidation could thus guide clinical precision treatment. Targeted treatments to specific regulatory molecules, such as *IPO5*, make personalized cancer therapy possible. Many oncogenic cellular processes can intervene effectively, which is the promise of accuracy in eradicating cancer and better patient care. Defining high-risk factors for developing CRCLM, stratification of high-risk individuals and minimization of the controllable risk factors are essential to prevent CRCLM and further reduce CRC mortality.

## Data availability statement

All the processed data are in the manuscript's supplementary data. Unfortunately, the raw data supporting the conclusions of this article can not be published in the public repository due to the lack of patient consent. Still, they will be made available by the authors on request. The validation data set was publicly available here GSE62321.

## Ethics statement

The studies involving human participants were reviewed and approved by ethics committees at the Faculty Hospital in Hradec Kralove, Czech Republic (number of approval 201207- S01P) and the Institute of Experimental Medicine, Prague, Czech Republic (number of approval 2018/05). The patients/participants provided their written informed consent to participate in this study.

## Author contributions

VV, JP, and LV secured the financial funding, VV and SF were responsible for the experimental design, OK, SJ, MM, and JP collected the patients’ samples, MK was responsible for the pathological assessment of the samples, AS performed the isolation of the samples and preparation for sequencing methods, KH and IC performed the methylation array analyses, JH and SG-M were responsible for bioinformatical analyses of data from all methods, VV, JH, and OK wrote the paper with the input from all authors. All co-authors have participated in the planning and execution of the study as well as the preparation of the manuscript. All authors contributed to the article and approved the submitted version.

## Supplementary material

Supplementary Figure 1 Descriptive mutational analysis **(A)** The distribution of genetic variation in percent according to the putative effect across all samples. Each column represents a patient. **(B)** The distribution of genetic variation in percent according to the single nucleotide variation effect across all samples. Each column represents a patient. **(C)** The distribution of genetic variation in percent according to PolyPhen2 across all samples. Each column represents a patient. **(D)** The distribution of genetic variation in percent across all samples after stratification for chromosome localizations. Each column represents a patient.

Supplementary Figure 2 Descriptive mutational analysis between SmCRC and MmCRC patients **(A)** The distribution of genetic variation in percent according to the putative effect between SmCRC and MmCRC patients. Each column represents a group either of SMCRC or MmCRC group of patients. **(B)** The distribution of genetic variation in percent according to the single nucleotide variation effect between SmCRC and MmCRC patients. Each column represents either of SMCRC or MmCRC group of patients. **(C)** The distribution of genetic variation in percent according to PolyPhen2 between SmCRC and MmCRC patients. Each column represents either of SMCRC or MmCRC group of patients. **(D)** The distribution of genetic variation in percent between SmCRC and MmCRC patients after stratification for chromosome localizations. Each column represents either of SMCRC or MmCRC group of patients.

Supplementary Figure 3 Mutational signatures **(A)** Mutational signature analysis in all samples. Each row is represented by a patient. **(B)** The distribution of mutational signatures in percent across all samples. Each column represents a patient.

Supplementary Figure 4 Densities of structural variants **(A)** The density of genomic sizes of structural variants in all patients. **(B)** The density of genomic sizes of structural variants in SmCRC patients. **(C)** The density of genomic sizes of structural variants in MmCRC patients.

Supplementary Figure 5 Genomic distribution of differentially methylated regions **(A)** The principal component analysis among metastasis (M) and adjacent liver tissue (H). **(B)** The Circos-Manhattan plot shows the CpG sites differentially methylated in CRCLM tissue compared to adjacent liver tissue, the x-axis shows the location in genome, and y-axis is -log(p-value). **(C)** The distribution of differentially methylated CpG sites in percent in relation to the position in gene. Profile of all sites, significantly hypermethylated and significantly hypomethylated, is shown. **(D)** The distribution of differentially methylated CpG sites in percent in relation to the CpG island position. Profile of all sites, significantly hypermethylated and significantly hypomethylated, is shown.

Supplementary Figure 6 Functional pathway enrichment analysis in KEGG **(A)** Emapplot of the gene set enrichment analysis (GSEA) of the significantly deregulated pathways in the Reactome terms (adj. p-value < 0.05, all 27 terms), **(B)** The overrepresentation analysis (ORA) of significantly up-regulated genes (adj. p-value < 0.05; all 15 terms), **(C)** The overrepresentation analysis (ORA) of significantly down-regulated genes (adj. p-value < 0.05; all 17 terms).

Supplementary Figure 7 Descriptives of miRNAs based results and KEGG based enrichments **(A)** The alignment of miRNA and other small non-coding RNAs (sncRNAs) sequences for each patient. Each column represents metastasis (M) and adjacent liver tissue (H), and the y-axis represents the number of reads to the sncRNAs groups. **(B)** The KEGG enrichment analysis of the top 10 differentially up-regulated miRNAs. **(C)** The KEGG enrichment analysis of the top 10 differentially down-regulated miRNAs.

Supplementary Figure 8 The Reactome *IPO5* gene interaction Emapplot of the overrepresentation analysis (ORA) of the interacted pathways in the Reactome terms for *IPO5* gene extracted from DepMap (adj. p-value < 0.05, top 50 terms).
