# Supplementary figures and images for: Differences in genome, transcriptome, miRNAome, and methylome in synchronous and metachronous liver metastasis of colorectal cancer
Source: Front Oncol. 2023 Apr 27;13:1133598. doi: 10.3389/fonc.2023.1133598 (PMC10172672; doi:10.3389/fonc.2023.1133598)

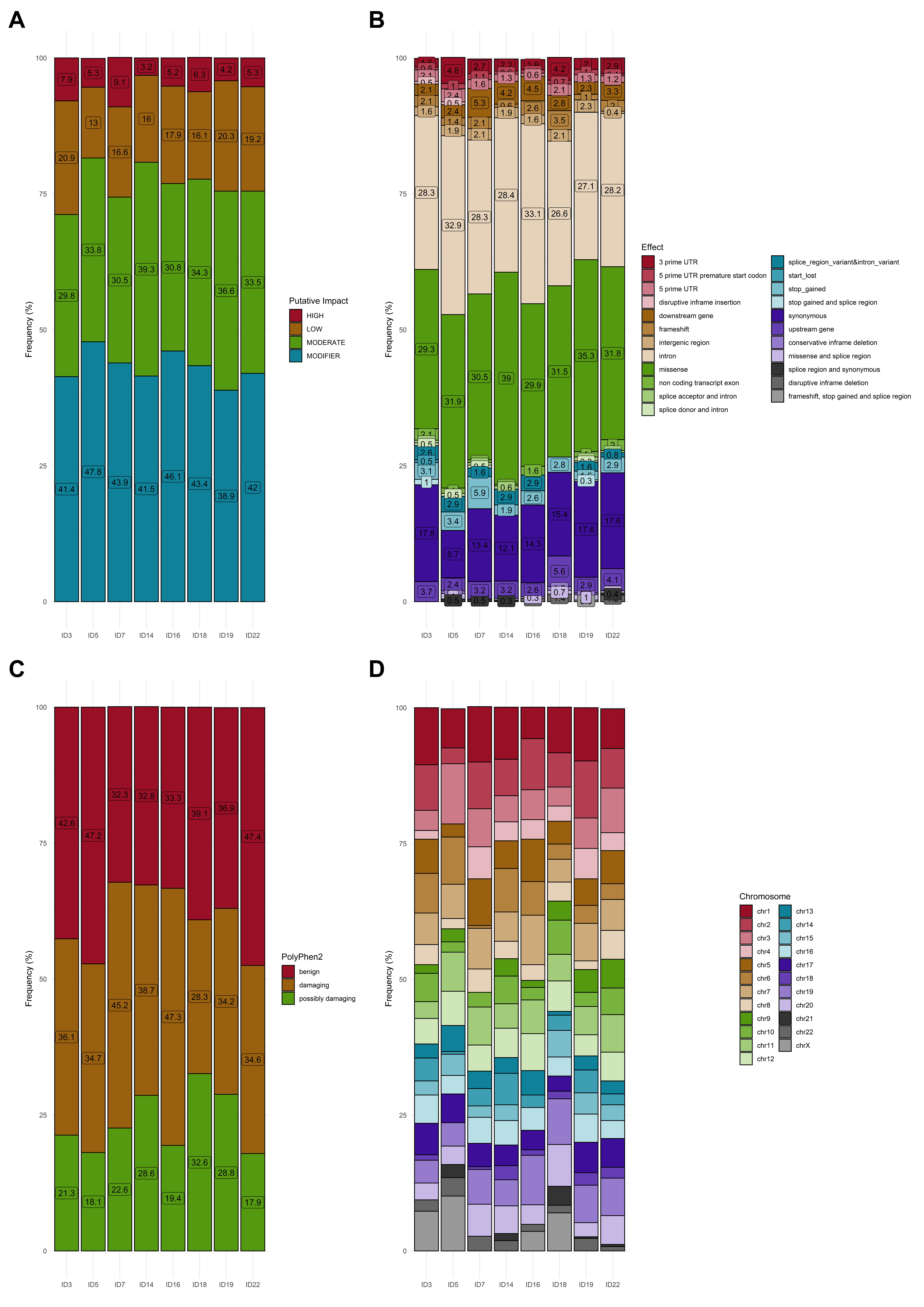

Supplement: Supplementary Figure 1 — Descriptive mutational analysis (A) The distribution of genetic variation in percent according to the putative effect across all samples. Each column represents a patient. (B) The distribution of genetic variation in percent according to the single nucleotide variation effect across all samples. Each column represents a patient. (C) The distribution of genetic variation in percent according to PolyPhen2 across all samples. Each column represents a patient. (D) The distribution of genetic variation in percent across all samples after stratification for chromosome localizations. Each column represents a patient. [file Image_1.jpeg]

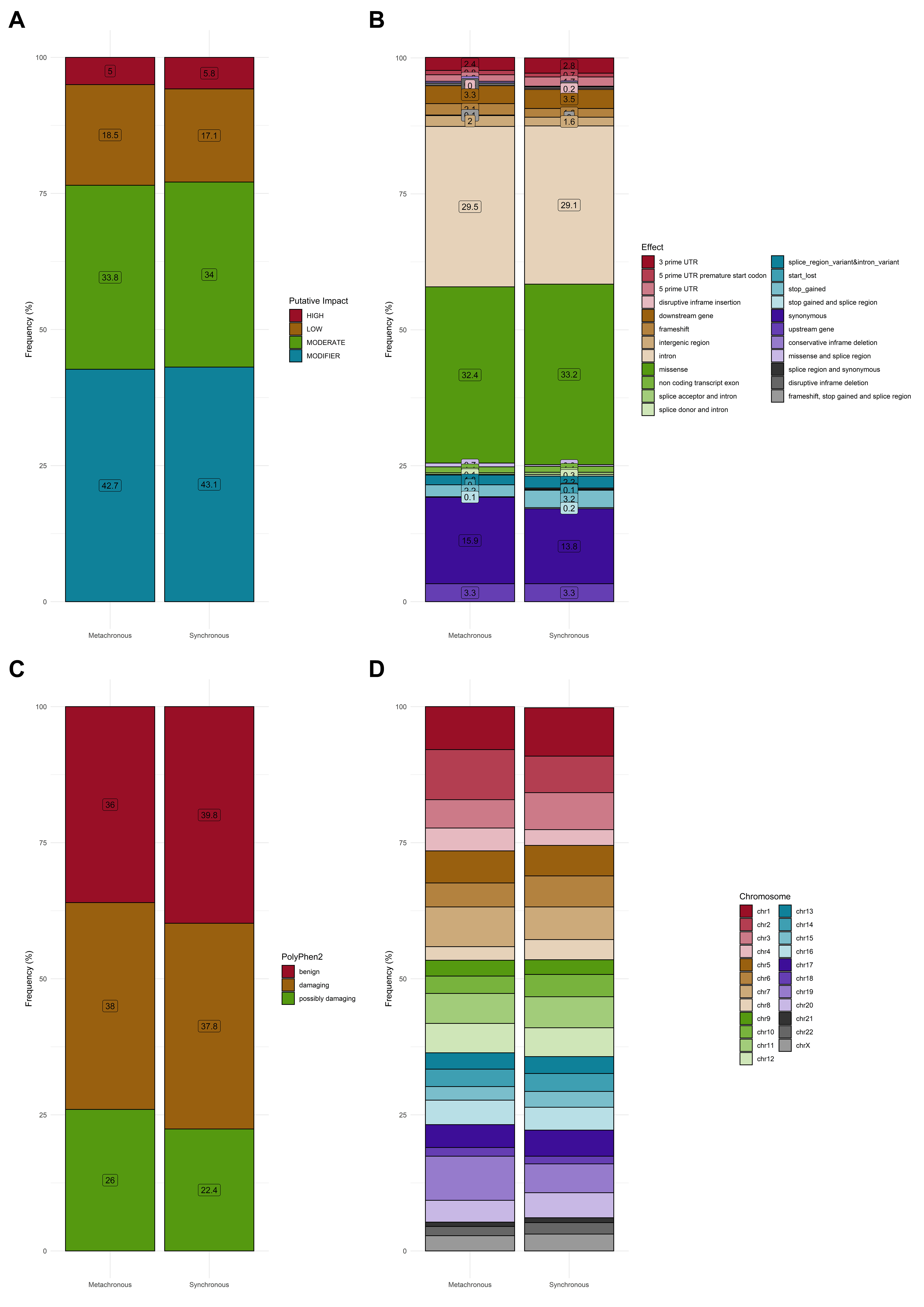

Supplement: Supplementary Figure 2 — Descriptive mutational analysis between SmCRC and MmCRC patients (A) The distribution of genetic variation in percent according to the putative effect between SmCRC and MmCRC patients. Each column represents a group either of SMCRC or MmCRC group of patients. (B) The distribution of genetic variation in percent according to the single nucleotide variation effect between SmCRC and MmCRC patients. Each column represents either of SMCRC or MmCRC group of patients. (C) The distribution of genetic variation in percent according to PolyPhen2 between SmCRC and MmCRC patients. Each column represents either of SMCRC or MmCRC group of patients. (D) The distribution of genetic variation in percent between SmCRC and MmCRC patients after stratification for chromosome localizations. Each column represents either of SMCRC or MmCRC group of patients. [file Image_2.jpg]

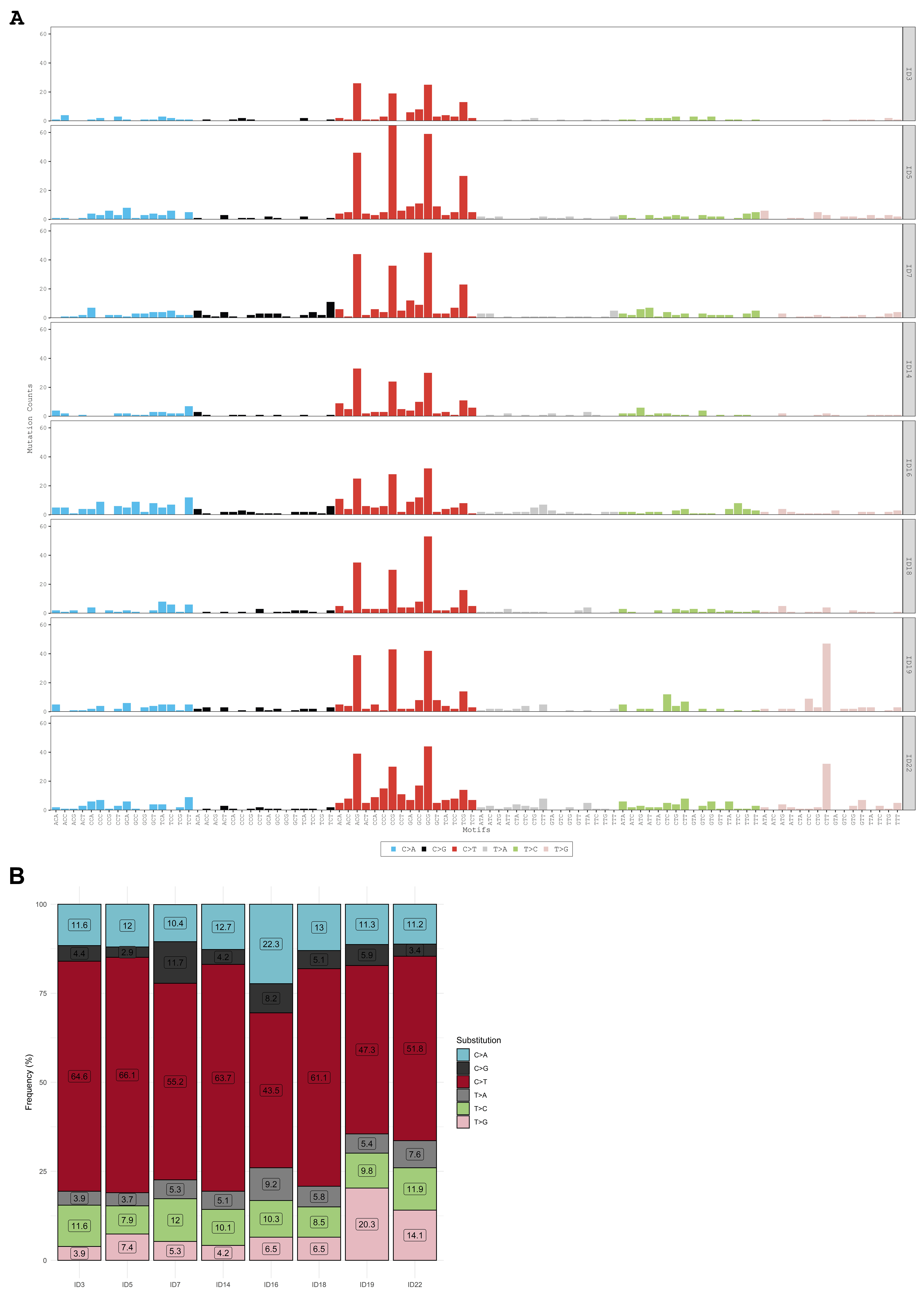

Supplement: Supplementary Figure 3 — Mutational signatures (A) Mutational signature analysis in all samples. Each row is represented by a patient. (B) The distribution of mutational signatures in percent across all samples. Each column represents a patient. [file Image_3.jpg]

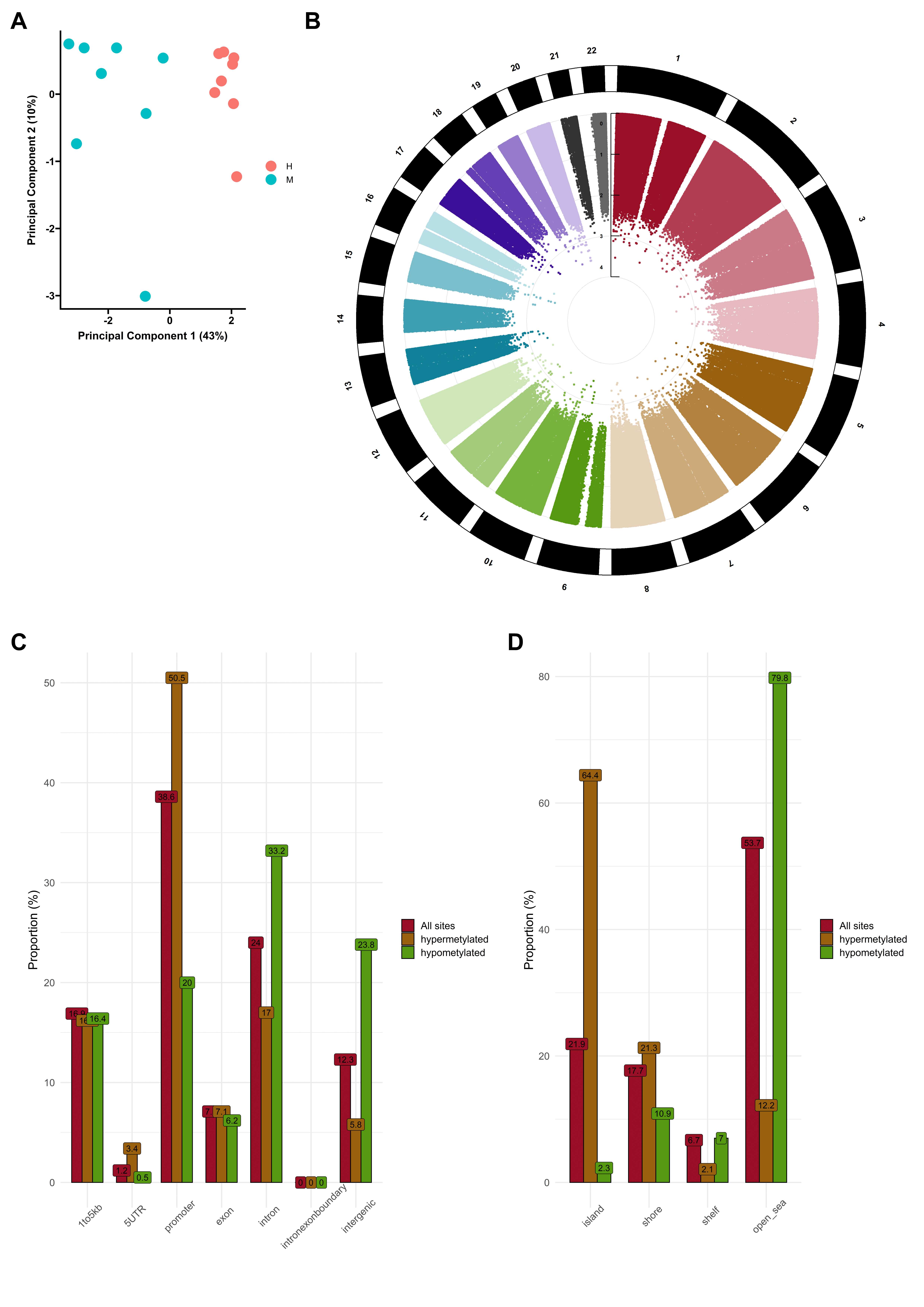

Supplement: Supplementary Figure 5 — Genomic distribution of differentially methylated regions (A) The principal component analysis among metastasis (M) and adjacent liver tissue (H). (B) The Circos-Manhattan plot shows the CpG sites differentially methylated in CRCLM tissue compared to adjacent liver tissue, the x-axis shows the location in genome, and y-axis is -log(p-value). (C) The distribution of differentially methylated CpG sites in percent in relation to the position in gene. Profile of all sites, significantly hypermethylated and significantly hypomethylated, is shown. (D) The distribution of differentially methylated CpG sites in percent in relation to the CpG island position. Profile of all sites, significantly hypermethylated and significantly hypomethylated, is shown. [file Image_5.jpeg]

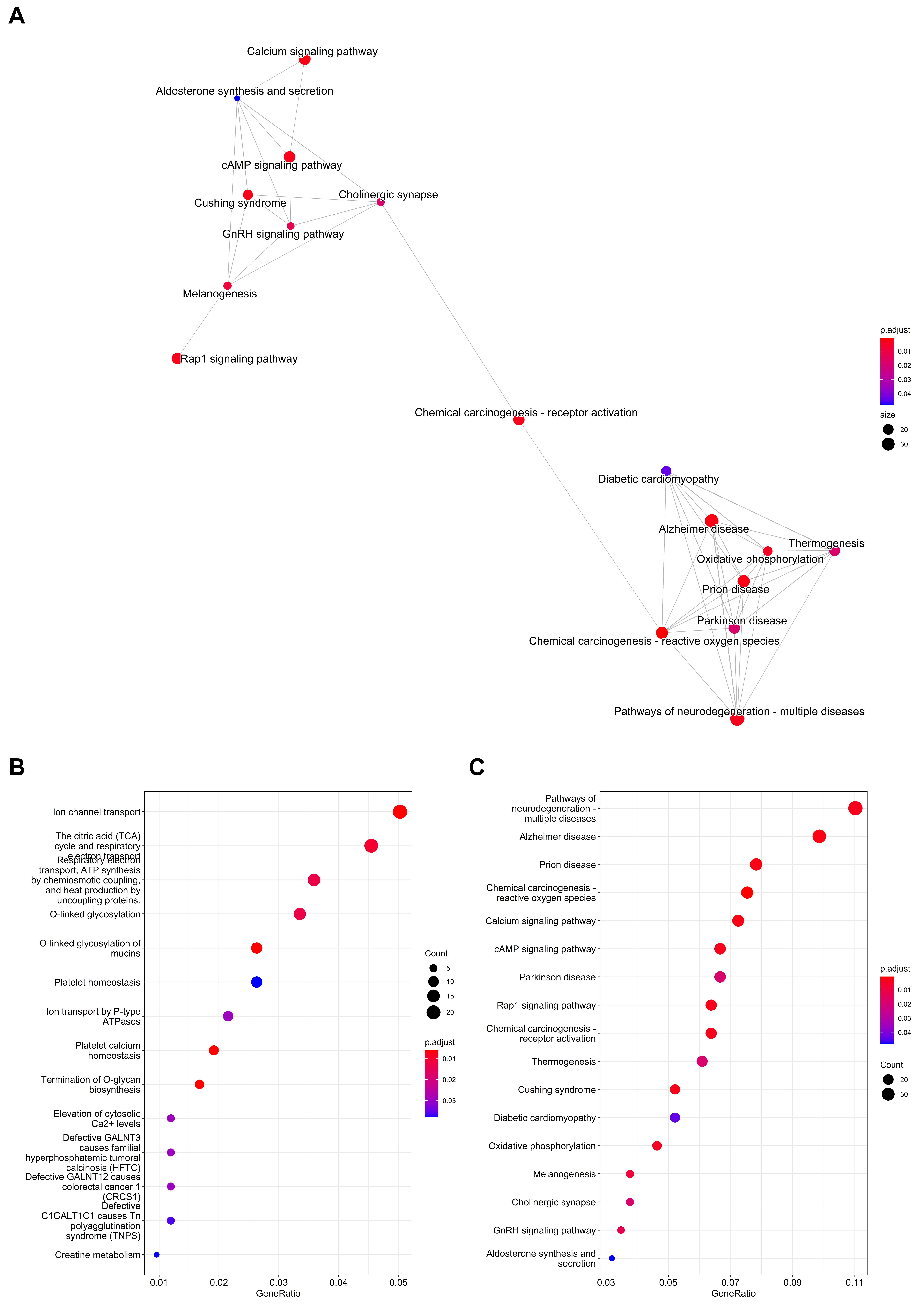

Supplement: Supplementary Figure 6 — Functional pathway enrichment analysis in KEGG (A) Emapplot of the gene set enrichment analysis (GSEA) of the significantly deregulated pathways in the Reactome terms (adj. p-value < 0.05, all 27 terms), (B) The overrepresentation analysis (ORA) of significantly up-regulated genes (adj. p-value < 0.05; all 15 terms), (C) The overrepresentation analysis (ORA) of significantly down-regulated genes (adj. p-value < 0.05; all 17 terms). [file Image_6.jpg]

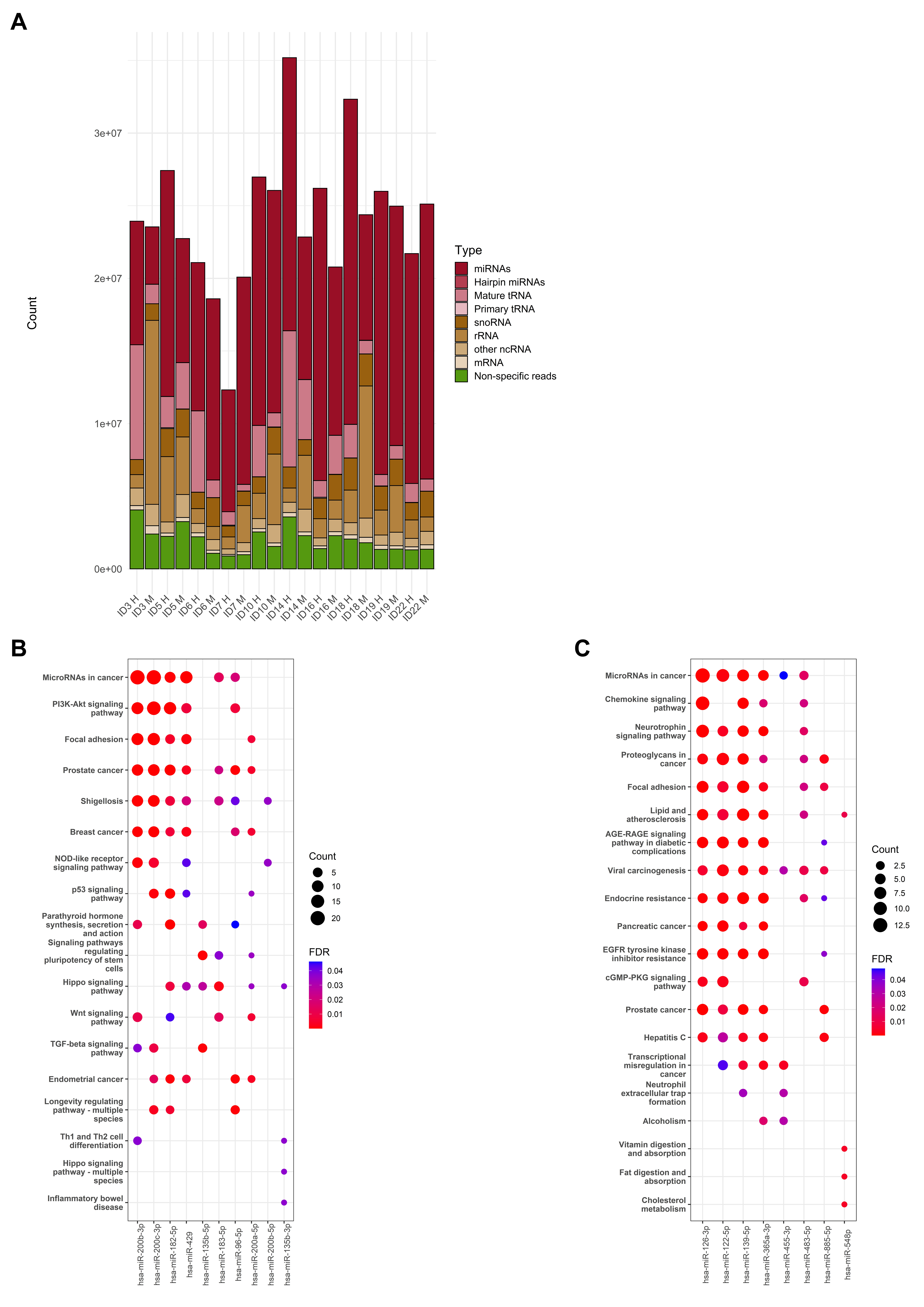

Supplement: Supplementary Figure 7 — Descriptives of miRNAs based results and KEGG based enrichments (A) The alignment of miRNA and other small non-coding RNAs (sncRNAs) sequences for each patient. Each column represents metastasis (M) and adjacent liver tissue (H), and the y-axis represents the number of reads to the sncRNAs groups. (B) The KEGG enrichment analysis of the top 10 differentially up-regulated miRNAs. (C) The KEGG enrichment analysis of the top 10 differentially down-regulated miRNAs. [file Image_7.jpg]

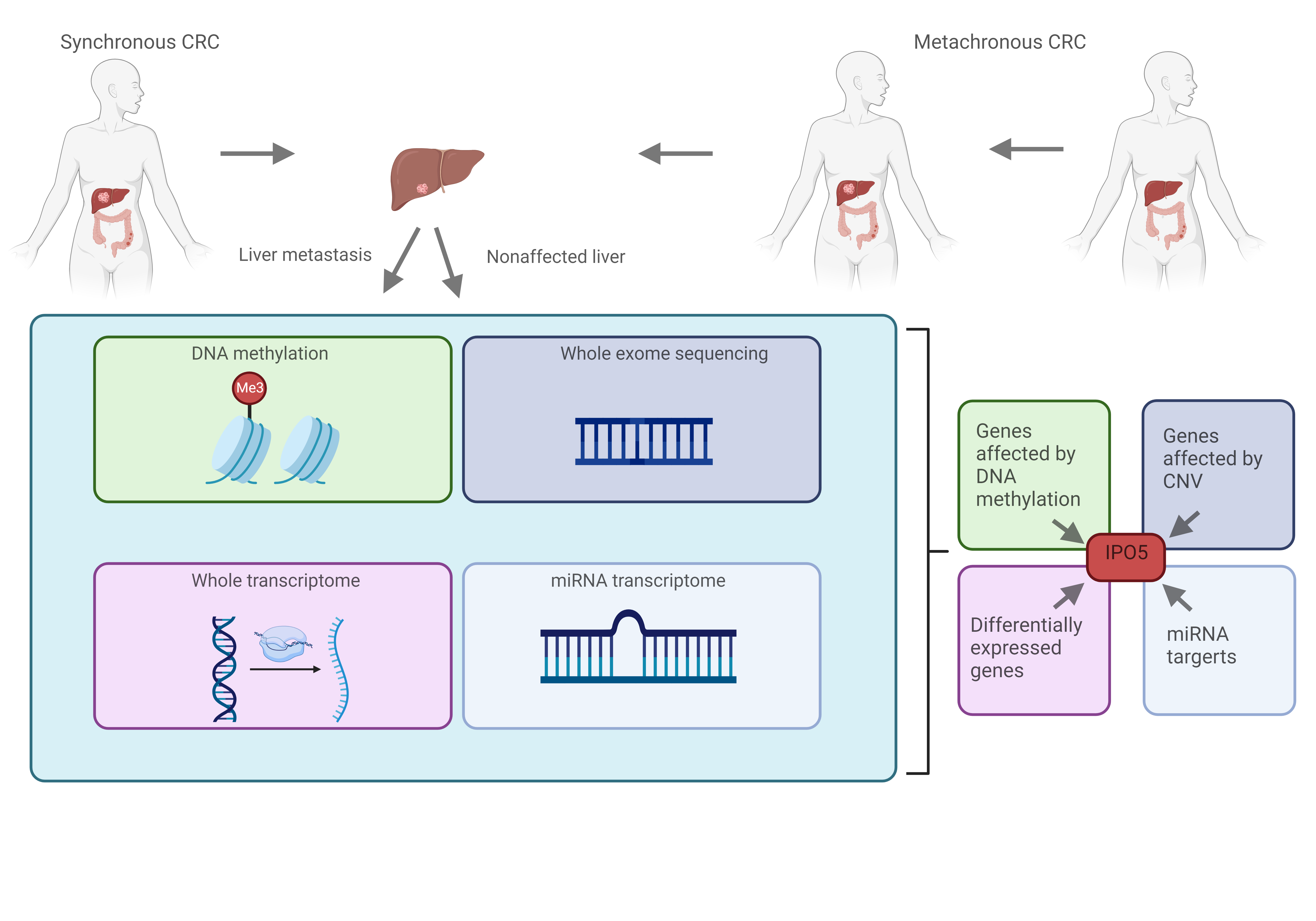

Supplement: Supplementary Figure 8 — The Reactome IPO5 gene interaction Emapplot of the overrepresentation analysis (ORA) of the interacted pathways in the Reactome terms for IPO5 gene extracted from DepMap (adj. p-value < 0.05, top 50 terms). [file Image_8.jpeg]
