# Supplementary material for: Differences in genome, transcriptome, miRNAome, and methylome in synchronous and metachronous liver metastasis of colorectal cancer
Source: Front Oncol. 2023 Apr 27;13:1133598. doi: 10.3389/fonc.2023.1133598 (PMC10172672; doi:10.3389/fonc.2023.1133598)
Supplement: Supplementary file 9 [file DataSheet_1.zip › MM_appendix R1.docx]

**A broader description of Materials and Methods**

*Sample collection, transport, and storage*

In this prospective single-center study, histologically confirmed CRCLM patients were recruited at the Department of Oncology and Radiotherapy (Hradec Kralove, Czech Republic) between June 2019 and December 2021. All CRCLM cases were monitored regularly until May 31st, 2022. Patients with any personal history of previous other malignancy or with CRC-associated well-defined inherited syndromes (including Lynch syndrome, familial adenomatous, and MUTYH-associated polyposis) were excluded from the study. In total, 10 CRCLM patients were included in the study.

CRCLM (n=10) and adjacent liver tissue (n=10) samples were collected during planned surgery. Considering the timing of the occurrence of liver metastasis, we could distinguish two groups of CRCLM patients: i) group of SmCRC (n=7) and ii) MmCRC (n=3). SmCRC cancers were classified as patients diagnosed with stage IV CRC, while MmCRC cancers were those who developed CRCLM after curing a primary CRC tumor. Regarding chronicity, we defined SmCRC and MmCRC cancers based on Moertel's definition. SmCRC cancers were defined as those occurring within three months from the first primary cancer, while MmCRC cancers were defined as those with an occurrence after three months postoperatively.

After collection, biological samples were immediately put in an RNA Later stabilization reagent (Invitrogen, USA) at the Department of Surgery (Hradec Kralove, Czech Republic), transferred in liquid nitrogen to The Fingerland Department of Pathology (Hradec Kralove, Czech Republic) stored at − 80 °C. All samples were then shipped to the Institute of Experimental Medicine (Prague, Czech Republic) for nucleic acid isolation and processing.

Biological specimens and clinical data were collected from all subjects recruited in the study. The following data were collected: gender, age at initial cancer diagnosis, tumor stage, CRC histology, and patients’ survival from the time of the first cancer diagnosis. The tumor stage of the CRC was determined according to Tumor-Node-Metastasis (TNM) classification. The clinicopathological data for the patients recruited are reported in Table 1.

The local ethics committees at the Faculty Hospital in Hradec Kralove, Czech Republic (number of approval 201207- S01P) and the Institute of Experimental Medicine, Prague, Czech Republic (number of approval 2018/05) approved the study. All patients provided written informed consent.

For correction of bioinformatic data (see below), the colon mucosa from healthy individuals was used. Fresh frozen samples were collected consecutively during the planned colonoscopy.

*Seed and soil mechanism and correction of bioinformatical data*

A small subset of CRC cells acquires the ability to escape from the primary CRC, in part by morphological changes, such as epithelial-to-mesenchymal transition (EMT), migration through the extracellular matrix (ECM), and invasion into the neighboring tissues, intravasation, survival in the circulation, extravasation and finally colonization to distant liver forming more aggressive secondary CRCLM.

The first cells invading the liver came from the primary CRC tumor. They thus also carry the genome/transcriptome of the primary tumor, respectively colorectum. Thus, comparing the genetic/molecular background of CRC cells with non-tumor liver cells could lead to results focused on organ specificity rather than on the distinctiveness of the metastasis relative to the healthy tissue. For this reason, we included a set of control colon tissues into the analyses apart from the patients’ paired samples (metastases and adjacent liver tissue). Overall, control colorectal tissues (n=10) were used to eliminate false positive results from contamination of liver tissue. These tissues were used from healthy individuals with no previous or current cancer. This correction was used for DNA methylation assessment and RNA seq analysis. A more detailed description of this correction is explained in the bioinformatics section of the methods.

*DNA-based methods*

*DNA isolation, quality, and quantity analyses*

Deep-frozen (-80°C) tissue samples were homogenized by MagNa Lyser Green Beads using MagNALyser Instrument (both La Roche, Germany). DNA from liver metastatic (i.e. CRCLM) and adjacent liver sections was extracted using a DNA Mini Kit (Qiagen, Germany) according to the manufacturer’s instructions and stored at -20°C. DNA concentration and quality were measured with a Qubit fluorimeter (DNA Broad Range assay, Qubit 3.0, Thermo Fisher Scientific, USA).

*Whole exome sequencing (WES)*

*Enrichment of DNA spanning exonic sequences from DNA*

We enriched protein-coding DNA using Human Whole Exome kit v7 (Agilent, USA) according to the manufacturer´s instructions.

*Library preparation*

DNA samples from CRCLM and adjacent liver tissues were sheared using the CovarisM220 ultrasonicator (Covaris, USA). Exome enrichment libraries were prepared using the SureSelect XT HS Library Preparation and SSELXT2 Human All Exon V7 Kits (both Agilent, USA) according to the manufacturer’s instructions. All 20 samples were pooled together and DNA fragments of 400 nt were sequenced using the paired-end mode, yielding 2x150 nt long reads on a NovaSeq 6000 (Illumina, USA). The median coverage was 109 (individual). The per-sample median value was calculated as a median of medians for every region covered by the exome enrichment probes.

*DNA sequencing data analysis*

The quality of raw reads was evaluated using FastQC (v0.11.9) and MultiQC (1.12). Reads that did not meet the defined standards (Quality Phred score cutoff: 30, Minimum required sequence length >36nt, clipping Illumina adaptor sequences, trimming the first 10nt) were removed, trimmed, or edited using the Trimmomatic tool (version 0.39).

Alignment to the human genome reference sequence (hg38 without decoy sequences) was performed using Burrows-Wheeler Aligner (BWA, v0.7.17-r1188) in default settings for all WES data. PCR duplicates were removed using PicardTools (v4.2.0) with the optical distance parameter set to 100 (the default).

For variant calling and filtering, we used the GATK software (v4.2.0.0) and its best practices pipeline “Somatic short variant discovery”. Tumor purity was assessed by the PureCN R package as well as the Copy Number Variation (CNV) calling. Variants were annotated, and their effect was predicted using SnpEff (5.0e) based on the Human genome database UCSC hg38.

Currently, there is no standard way to calculate Tumor mutational burden (TMB). Some studies excluded synonymous mutations, while others included them. We defined all mutations (aTMB), which include synonymous mutations, and fTMB, which exclude synonymous mutations per megabase (Mb) of the sequenced region.

*DNA methylation analysis*

DNA (1000 ng) was treated overnight with sodium bisulfite using the EZ DNA Methylation Kit (Zymo Research, USA) to convert unmethylated cytosines to uracils while methylated cytosines remained unchanged. The bisulfite-converted DNA (BCD) samples were stored at −80 °C until use. BCD was processed using the Infinium Methylation EPIC Kit (Illumina, USA) according to the manufacturer’s protocol (Infinium HD Methylation Assay Protocol), including enzymatic fragmentation, precipitation, and hybridization, followed by BeadChip washing and staining. The chips allowed the detection of over 850,000 methylation sites per sample across the genome at single-nucleotide resolution. The methylation status at each CpG site, scanned by the iScan System (Illumina, USA), was estimated by measuring the intensity of the pair of methylated and unmethylated probes.

*Processing of methylation data*

Beta values for determining the methylation level as the ratio of the fluorescent signals from the methylated vs. unmethylated sites were calculated using the minfi package. Preprocessing analyses were performed to study the distribution of beta values and the methylation variation across all samples.

Raw microarray data were downloaded as idat files, imported to the R environment, and processed with the minfi package. Data were normalized using the quantile method. A series of filtering was performed. Probes with single nucleotide polymorphisms (SNPs) at CpG sites and the cross-reactive probes were excluded, resulting in 794,441 probes. The last filtering step was performed due to the necessity to discard all probes significantly changed between the colon and liver tissue (significance calculated using the same approach described below) to eliminate false positive results from liver tissue contamination and resulting in the final number of 559,364 probes. We estimated associations between principal components and slide factors and used the Combat function (sva package) for batch correction.

Principal component analysis (PCA) was performed to identify the variance using the covariance matrix. We identified differentially methylated loci using the top Table function (limma package). For multiple testing of the false discovery rate (FDR), the p-values for the contrast of interest were adjusted to be below <0.01, which is regarded to be the most appropriate for microarray analysis.

The proportions of genomic regions to gene positions were analyzed using the annotatr package. Annotation of the CpG site to ENTREZID, plots of the Kyoto Encyclopedia of Genes and Genomes (KEGG) and Reactome pathways, and an enrichment map were obtained using clusterProfiler v4.0 and ReactomePA packages.

*RNA-based methods*

*RNA isolation, quality, and quantity analyses*

Total RNA, including the long noncoding RNA (lncRNA) fraction, was isolated from deep-frozen (-80°C) tissue samples with an RNeasy kit (Qiagen, Germany) using the one-column protocol according to the manufacturer’s recommendation. All tissues were homogenized by MagNa Lyser Green Beads using MagNALyser Instrument (both La Roche, Germany). RNase-Free DNase Set (Qiagen, Germany) was used for DNase treatment of RNA samples. RNA concentration was measured on a Qubit fluorometer 3.0 with the Qubit™ RNA HS and BR Assay Kit (Invitrogen, USA) and on the NanoDrop-1000 instrument (Thermo Fisher Scientific, USA) to determine the purity values (OD260/280, OD260/230). RNA quality analysis was performed on an Agilent Bioanalyzer 2100 microcapillary electrophoresis system with the RNA 6000 Nano Kit (both Agilent, USA). Samples with RNA Integrity Number (RIN) ≥ 8 were used for subsequent analysis.

*Library preparation*

An aliquot with 1000 ng of total RNAs was used for rRNA removal and subsequent RNA-seq library preparation. Ribosomal RNAs (rRNAs) were removed using NEBNext rRNA Depletion Kit (Human/Mouse/Rat, New England Biolabs, USA). All RNA sequencing cDNA libraries were constructed according to the NEBNext Ultra II Directional RNA Library Prep Kit for Illumina, as provided by the manufacturer (New England Biolabs, USA).

To validate the quality and to assess the size distribution of the cDNA library, an aliquot was loaded onto an Agilent High Sensitivity DNA chip and run on Agilent Bioanalyzer 2100 High sensitivity DNA Kit (Agilent, USA). The samples were analyzed using the KAPA Library Quantification Kit for Illumina, a fluorometric-based system (Thermo Fisher Scientific, USA) for accurately quantifying the DNA library. The cDNA libraries were pooled, and an aliquot was loaded into a High Output flow cell and sequenced paired-end (2 × 150 bp) on NovaSeq 6000 (Illumina, USA).

*RNA-seq data analysis*

The quality of raw reads was evaluated using FastQC (v0.11.9), MultiQC (1.12), and reads that did not meet the defined standards (Quality Phred score cutoff: 30, Minimum required sequence length >36nt, clipping Illumina adaptor sequences) were removed, trimmed, or edited using Trimmomatic tool (version 0.39). Ribosomal RNA was filtered using the BBDuk tool from BBMap (v38.90).

Clean reads were aligned to the human genome reference sequence (hg38 without decoy sequences) by STAR aligner (v2.7.10a) in the default setting. Quantification of gene expression was performed using RSEM (v1.3.3) in default settings. The tumor purity, immune and stromal proportion, and contamination of liver tissue were assessed by the ratio of somatic variants in non-differently expressed genes and by the ESTIMATE R package ahead of the final expression calculation. The normalized counts of metastatic samples were then cleaned from the contamination of liver tissue by subtracting the calculated proportion of the liver tissue normalized counts. Using the likelihood ratio test, significantly differentially expressed genes (DEGs) between CRCLM and non-malignant colon tissue were identified with EdgeR (v3.38.0) R package. After Benjamin Hochberg adjustment (BH), the gene was considered deregulated (DE) with a false discovery rate lower than 0.05.

Functional enrichment analysis was performed using the clusterProfiler, and ReactomePA R packages. Only terms associated with a BH-adjusted p-value lower than 0.05 were considered.

*microRNA(miRNA)-based methods*

*miRNA isolation, quality, and quantity analyses*

Deep-frozen (-80°C) tissue samples were homogenized using MagNA Lyser Green Beads in the MagNA Lyser Instrument (both La Roche, Germany). Total RNA, including miRNA, was isolated using mirVana™ miRNA Isolation Kit without miRNA enrichment (Thermo Fisher Scientific, USA). The quality and concentration of miRNA were analyzed by Agilent Bioanalyzer 2100 (Agilent, USA), using the Small RNA Analysis Kit (Agilent, USA). Qubit 3.0 Fluorometer and Qubit dsDNA HS Assay Kit (Thermo Fisher Scientific, USA) were used to control miRNAs’ quality.

*Small RNA-Sequencing*

MiRNA libraries were constructed using the NEB Next Multiplex Small RNA Library Prep Set for Illumina (New England BioLabs, USA) according to the manufacturer’s protocols. Briefly, miRNA samples (5-10 ng) were ligated with 5´and 3´adapters, followed by reverse transcription- for cDNA library construction and incorporation of index tags. The cDNA library fragments were purified by AMP Pure XP Beads (Beckman Coulter, USA) and separated on a 6% TBE PAGE gel (Thermo Fisher Scientific, USA), and 145–160 bp size fraction containing miRNA inserts was isolated. The twenty cDNA library samples were pooled in equimolar amounts and used for cluster generation and sequence analysis in a single lane on an Illumina NovaSeq6000 (50 bp single read).

The quality of raw reads was evaluated using FastQC (v0.11.9) and MultiQC (1.12) and reads that did not meet the defined standards (Quality Phred score cutoff: 30, Minimum required sequence length >16nt, clipping Illumina adaptor sequences) were removed, trimmed or edited using Cutadapt tool (v3.4). rRNA was filtered out using the BBDuk tool from BBMap (v38.90).

The miRge3.0 pipeline was used for alignment and quantification. It integrates the Bowtie tool for alignment and performs the first alignment against the microRNA database MiRBase, followed by the second alignment against the hg38 reference genome for unmapped reads from the first alignment. The likelihood ratio test identified Significantly different miRNAs with EdgeR (v3.38.0) R package. After BH adjustment, the miRNA was considered DE with a false discovery rate lower than 0.05.

*External Validation*

For external validation of our data, the GSE62321 set was used 48,49. This set comprises 57 samples of patients with stage IV colorectal cancer as well as pairs of primary tumors and hepatic metastases before chemotherapy from 13 patients.

*Bioinformatic in silico analysis*

*DepMap data analysis*

To analyze the IPO5 gene interactions, the correlation data were downloaded from the CRISPR (Avana) Public Depmap v20Q3 portal <https://depmap.org/portal/download/> for all cell lines in the database (1,078 cell lines). Gene correlation between *IPO5* knockout effect and gene expression was considered significant for p-value lower than 0.05 and absolute value of correlation coefficient higher than 0.1. Functional enrichment analysis of significantly correlated genes with *IPO5* effect was performed using the clusterProfiler, and ReactomePA R packages. Only terms associated with a BH-adjusted p-value lower than 0.05 were considered.
